# Mutations in a Guanylate Cyclase GCY-35/GCY-36 Modify Bardet-Biedl Syndrome–Associated Phenotypes in *Caenorhabditis elegans*

**DOI:** 10.1371/journal.pgen.1002335

**Published:** 2011-10-13

**Authors:** Calvin A. Mok, Michael P. Healey, Tanvi Shekhar, Michel R. Leroux, Elise Héon, Mei Zhen

**Affiliations:** 1 The Program in Genetics and Genome Biology, The Hospital for Sick Children, Toronto, Canada; 2 Samuel Lunenfeld Research Institute, Mount Sinai Hospital, Toronto, Canada; 3 Institute of Medical Science, University of Toronto, Toronto, Canada; 4 Department of Molecular Biology and Biochemistry, Simon Fraser University, Burnaby, Canada; 5 Department of Molecular Genetics, University of Toronto, Toronto, Canada; Duke University, United States of America

## Abstract

Ciliopathies are pleiotropic and genetically heterogeneous disorders caused by defective development and function of the primary cilium. Bardet-Biedl syndrome (BBS) proteins localize to the base of cilia and undergo intraflagellar transport, and the loss of their functions leads to a multisystemic ciliopathy. Here we report the identification of mutations in guanylate cyclases (GCYs) as modifiers of *Caenorhabditis elegans bbs* endophenotypes. The loss of GCY-35 or GCY-36 results in suppression of the small body size, developmental delay, and exploration defects exhibited by multiple *bbs* mutants. Moreover, an effector of cGMP signalling, a cGMP-dependent protein kinase, EGL-4, also modifies *bbs* mutant defects. We propose that a misregulation of cGMP signalling, which underlies developmental and some behavioural defects of *C. elegans bbs* mutants, may also contribute to some BBS features in other organisms.

## Author Summary

Bardet-Biedl syndrome (BBS) is a genetically heterogeneous, multisystemic disorder. Defects to the cilium, an evolutionarily conserved organelle, cause ciliopathies, a growing class of diseases that includes BBS. BBS proteins are involved in the vesicular transport of proteins to the cilium and in the process of intraflagellar transport. Here we show that, in addition to sensory defects, *Caenorhabditis elegans bbs* mutants exhibit reduced body size and delayed developmental timing. The reduced body size phenotype is not fully recapitulated by IFT mutants, suggesting that BBS proteins may have additional functions beyond bridging IFT motors. We further identified that the loss of function mutations in the soluble guanylate cyclase complex, GCY-35/GCY-36, results in a suppression of these defects. Interestingly, GCY-35/GCY-36 influences the body size through a cGMP-dependent protein kinase EGL-4 in a group of body cavity neurons. BBS proteins, on the other hand, function through a non-overlapping set of ciliated sensory neurons to influence cGMP signalling in the body cavity neurons. In conclusion, this study reveals a non-cell autonomous role for sensory cilia in regulating cGMP signalling during development. We propose that aberrant cGMP signalling, essential for a number of cellular processes, may also contribute to some ciliopathy features in other systems.

## Introduction

The cilium plays diverse cellular functions in metazoans which include imparting motility, enabling sensory processes and regulating the activity of cell signalling pathways during development [1]. The biogenesis and maintenance of this evolutionarily conserved organelle relies on intraflagellar transport (IFT) – the bidirectional transportation of diverse cargo proteins along the microtubule-based axoneme. Defective IFT or ciliary dysfunction result in ciliopathies, a growing class of pleiotropic human diseases with overlapping clinical features, some being of significant morbidity [2]. Bardet-Biedl syndrome (BBS, OMIM 209900) is an autosomal recessive and genetically heterogeneous ciliopathy with hallmark clinical features that include photoreceptor degeneration, renal abnormalities, obesity, cognitive impairment, and digit and genital anomalies [3]. To date, sixteen genes are associated with BBS [3]–[5]; of these, eight function mostly as a conserved protein complex (BBSome) [6] to regulate vesicular sorting and packaging [7], IFT [8]–[9], as well as cilium maintenance and function (reviewed in [10]).

Animal models have been instrumental in deciphering the physiological functions of BBS proteins [2], [10]. Initial characterization of *Caenorhabditis elegans* BBS orthologues led to the discovery of BBS proteins as ciliary components, associating ciliary defects with the loss of BBS protein function [11]–[13]. The loss of BBS-7 and BBS-8 led to shortened cilia, reduced uptake of a lipophilic dye (DiI) by the cilium, and defective chemo- and thermotaxis [13]–[14].

Murine *Bbs* mutants recapitulate several human BBS features including photoreceptor degeneration, renal anomalies and obesity [15]–[16]. Additionally, these models led to the identification of new features such as neural tube closure defects [17], anosmia [18], and behavioural, mechano- and thermosensory deficits [14] that expanded the diagnostic features of human ciliopathies. Morpholino-mediated knockdown of *bbs* in zebrafish led to developmental phenotypes such as dorsal thinning, poor somitic definition and Kupffer's Vesicle malformation [19]–[21], while defects characteristic of ciliopathy such as delayed retrograde melanosome transport [20] and vision impairment [22] also manifested.

In addition to a role in sensory transduction, the primary cilium functions as a signalling ‘apparatus’ to regulate development [1]. For example, IFT-dependent localization of Sonic Hedgehog (Shh) receptors to primary cilia is required for Shh signalling [23]–[24]. Disrupting IFT components IFT172, TG737/Polaris and the motor KIF3A in the mouse resulted in phenotypes typical of Shh mutants [25]. Similarly, defective planar cell polarity (PCP) signalling [17], [26] and/or aberrant Wnt signalling [27]–[28] were associated with the inactivation of BBS, Polaris or KIF3A components. These, and others studies [29]–[30] suggest that the cilium may modulate multiple signalling pathways in a tissue-specific manner. Aberrant PCP, Shh and Wnt signalling have been implicated in underlying a number of ciliopathy features, such as neural tube closure, polydactyly and obesity [17], [22], [31]–[32]. The pathology of other features such as photoreceptor degeneration, remains largely unexplained, indicating the presence of unidentified cellular processes that are regulated by the cilium.

*C. elegans* BBS orthologues are exclusively expressed by 60 ciliated neurons. Localizing at the base of cilia, they undergo active IFT, and their absence results in the destabilization of IFT and sensory defects [8]. *C. elegans* sensory neurons play key roles in multiple developmental processes [33]. Some chemosensory mutants exhibit a reduced body size [33], indicating that sensory function may influence this developmental process. Another key regulator for body size is the cGMP-dependent protein kinase (PKG) EGL-4 [33]–[36]; a loss of EGL-4 function leads to increased body size that is genetically epistatic to that of chemosensory mutants [33]. The mechanisms for sensory neuron-mediated body size regulation, however, remain to be fully elucidated. In the present study, in order to identify additional cilium-regulated signalling events in *C. elegans*, we carried out the phenotypic characterization of *bbs* mutant animals, and identified genetic modifiers that associate aberrant cGMP signalling with a subset of *bbs* features.

## Results

### *C. elegans bbs* mutants share ciliary defects, reduced body size, delayed developmental timing, and reduced roaming

**Figure 1:**
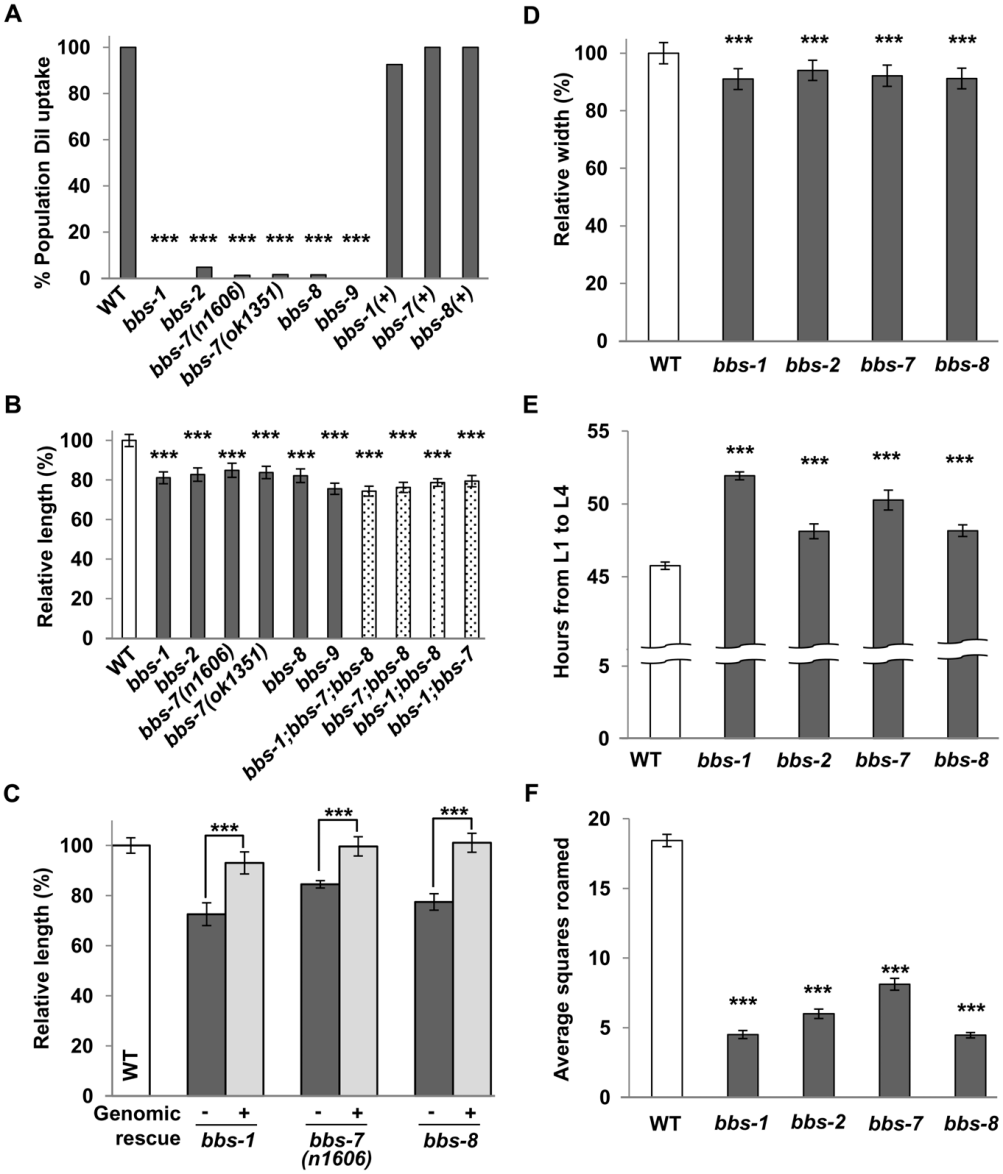
Multiple endophenotypes exhibited by *bbs* mutants. (A) DiI uptake of *bbs* mutants was reduced compared to wild-type and transgenic *bbs* mutants carrying genomic DNA fragments for respective *bbs* genes. Fisher's Exact test, *** p<0.001. (B) The body length of young adult *bbs* single, double and triple mutants was smaller in comparison to wild-type animals. ANOVA with Tukey, *** p<0.001, n≥20. Data represent mean ± SD normalized against wild-type body length. (C) The body length defects of *bbs* mutants were rescued by the expression of wild-type *bbs* genes (+) compared to their non-transgenic siblings (−). ANOVA with Tukey, *** p<0.001, n≥15. (D) The body width of *bbs* mutants was also decreased in comparison to wild-type animals. ANOVA with Tukey, *** p<0.001, n≥20. (E) Developmental timing (from L1 to L4 stage) was increased for *bbs* mutants in comparison to wild-type animals. ANOVA with Tukey, *** p<0.001, n≥50, N≥10 replicates. Data represent mean (hours) ± SD. (F) *bbs* mutants roamed less compared to wild-type animals. Kruskal-Wallis with Dunn's, *** p<0.001, n≥25, N≥2 replicates. Data represent mean squares roamed ± SEM. For *bbs-7* dataset, the *ok1351* deletion allele was used unless otherwise noted.

We performed a thorough phenotypic and behavioural assessment of severe or complete loss-of-function (*lf*) mutants for the *C. elegans bbs-1*, *-2*, *-7*, *-8* and *-9* genes (Table S1). Consistent with previous reports on *bbs-7* and *-8* [13], all examined *bbs* mutants exhibited a failure in the uptake of a lipophilic dye DiI by sensory neurons (Figure 1A), confirming a common structural and functional deficit in the sensory cilia. In addition to sensory defects, we identified three novel *bbs*-associated phenotypes: decreased body size, altered dwelling/exploration behaviour and delayed developmental timing.

Despite grossly normal body morphology, *bbs* mutants shared a reduced body length by ∼11–28% when compared to wild-type animals. Defects were visible during early larval stages and persisted throughout adulthood (Figure 1B and Figure S1). In these analyses, we defined a 3.5% or greater difference as biologically relevant in body size change, as this was the upper range for the coefficient of variance in young adult wild-type populations. The reduced body length is caused by the loss of BBS function, as it was fully rescued by introducing a wild-type genomic or cDNA copy of *bbs* into the respective mutants (Figure 1C). A decrease in body width was also characteristic of *bbs* mutants (Figure 1D). By DAPI staining of nuclei we did not observe differences in tissue and cell numbers between wild-type and *bbs-7* animals (data not shown). The overall decrease in body size is thus best attributed to a smaller, averaged cell size in *bbs* mutants.

*bbs* double and triple mutants exhibited smaller body sizes that were no more severe than the smallest *bbs* single mutant. Although small differences between the body length of *bbs* single, double and triple mutant strains were observed as animals aged, the effects, however, were not additive (Figure 1B), consistent with BBS proteins functioning in the same biological processes to regulate ciliary development and function [6]–[7], [37].

We further examined the body size of a number of IFT mutants, including *che-2*, *-3*, and *-11*, as well as *osm-3*, *-5*, *-6* and *klp-11*, all of which display defects to cilia structure and abnormal dye filling (*dyf*). Only some exhibited decreases in body size; among them, *che-11* mutants exhibited the most significant decrease (by 11% in young adulthood), but not as severely as in age-matched *bbs* mutants (Figure S2A). Notably, a loss of the IFT motors, KLP-11 and OSM-3 kinesins, and CHE-3 dynein had little or no effect on body size. Therefore while sensory neurons affect body size, the *dyf* phenotype, caused by defective IFT transport, is not indicative of severe body size defects. The BBSome, exclusively expressed in ciliated sensory neurons, has a greater influence in the regulation of body size, indicating a role beyond bridging IFT motors for BBS proteins in these neurons.

*C. elegans* exhibits a defined developmental time course [38]. Multiple *bbs* mutant strains exhibited slower larval development (Figure 1E), resulting in a 2.3–6.2 hour delay between the first (L1) and last (L4) larval stage. During foraging, *C. elegans* exhibits a combination of dwelling and exploration/roaming behaviours that are altered in some chemosensory defective mutants [33]. Multiple *bbs* mutants showed a 56% to 76% decrease in overall movement, or roaming (Figure 1F) when compared to wild-type animals. This behavioural change does not reflect a general loss of locomotor activity, as *bbs* mutant animals exhibit normal locomotion during roaming. These additional phenotypes support a notion that the *C. elegans* cilia regulate cellular processes in addition to taxis behaviours.

### The loss of function of a guanylate cyclase subunit GCY-35 rescues a subset of endophenotypes in *bbs* mutants

**Figure 2:**
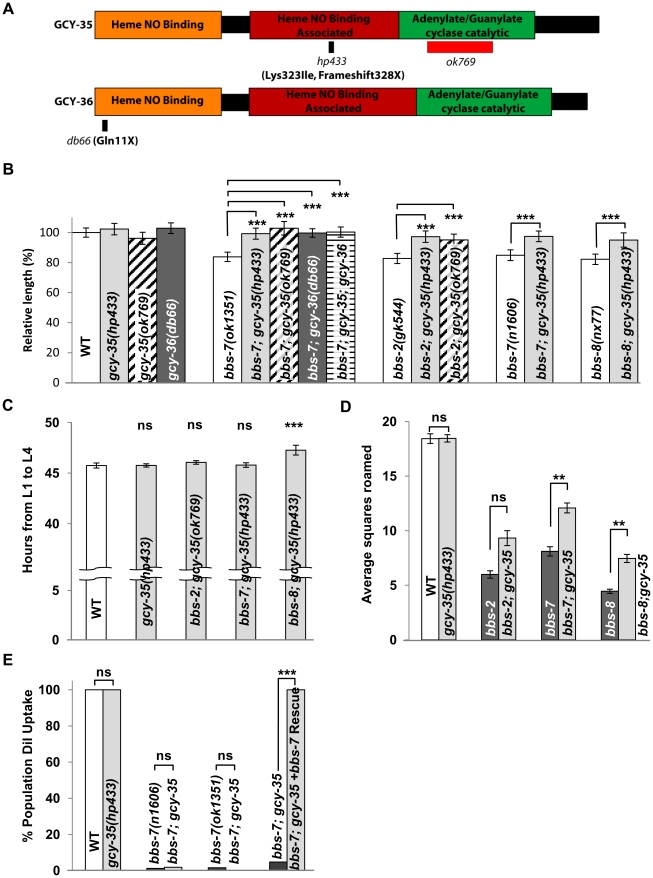
*gcy-35(lf)* suppresses a subset of *bbs* endophenotypes. (A) A schematic of the functional domains of GCY-35 and GCY-36 including allelic information of the respective mutants. (B) *gcy-35(lf)* and *gcy-36(lf)* alleles suppressed body length defects in multiple *bbs* mutant alleles. ANOVA with Tukey, *** p<0.001, n≥20. Data represent mean ± SD normalized against wild-type body length. (C) *gcy-35(lf)* fully or partially suppressed *bbs* mutant developmental defects. ANOVA with Tukey, *** p<0.001 with respect to wild-type controls; ns – p≥0.05, n≥50, N≥10 replicates. Data represent mean (hours) ± SD. (D) Exploration patterns of *gcy-35;bbs-7* and *gcy-35;bbs-8* animals (light bars) were partially rescued compared to respective *bbs* genotypes (dark bars). Kruskal-Wallis with Dunn's, ** p<0.01; ns – p≥0.05, n≥25, N≥2. Data represent mean squares roamed ± SEM. (E) *gcy-35* did not rescue the DiI uptake of *bbs-7* mutants, while *gcy-35(lf)* alone did not result in any DiI defects. Fisher's Exact test, *** p<0.001; ns – p>0.05. For *bbs-7* data depicted, the *ok1351* deletion allele was used unless otherwise noted.

Unlike all other *bbs* strains, MT3645 *bbs-7(n1606)* (received from the *Caenorhabditis Genetics Center)* displayed body size, roaming behaviour and developmental timing characteristics of wild-type animals. Upon genetic outcrossing, we re-isolated a homozygous *bbs-7(n1606)* strain that exhibited phenotypes characteristic of other *bbs* mutants. These defects were fully rescued by the expression of the wild-type genomic copy of *bbs-7* (Figure 1D). We concluded through genetic analyses that a single modifying locus from the original *MT3645* strain rescued a subset of the *bbs-7* mutant endophenotypes (Figure 2).

We mapped this modifier allele, *hp433*, to *gcy-35* (Materials and Methods), a gene encoding the α subunit of a soluble guanylate cyclase (sGC). sGC proteins are composed of a heme/NO binding (HNOB), a heme/NO binding associated (HNOBA), and a GC catalytic domain (reviewed in [39]). They are heterodimeric complexes consisting of α and β subunits to catalyze the conversion of GTP to cGMP. *hp433* results in a frame-shift in the coding sequence and a premature termination codon in the HNOBA domain, causing a truncation of the GC catalytic domain (Figure 2A). We also saw that a deletion allele in the GC domain of *gcy-35*, *ok769*, functions as a recessive suppressor of *bbs-7* mutants (Figure 2A and 2B).

While both *gcy-35*(*lf*) alleles exhibit similar body length to that of wild-type animals, they suppressed the significant body length defects of multiple *bbs* mutants (Figure 2B). In contrast, *gcy-35(lf);dyf* mutants showed little or no improvement to body length (Figure S2B). The consistent suppression observed in *bbs* mutant animals advocated strongly for the further investigation of *gcy-35(lf)* as an epistatic suppressor of *bbs*-mediated phenotypes. *gcy-35* also modified the *bbs* endophenotypes in developmental timing and roaming. Both the developmental timing from the first (L1) to the last (L4) stages, and roaming scores of *gcy-35* mutants were comparable to that of wild-type (Figure 2C and 2D). The developmental timing of *gcy-35;bbs-7* and *gcy-35;bbs-2* mutants was identical to that of wild-type animals, whereas *gcy-35;bbs-8* animals showed a partial improvement over that of *bbs-8* animals (Figure 2C). Roaming defects of *bbs-2*, *bbs-7*, and *bbs-8* mutants were partially suppressed by *gcy-35* (Figure 2D).

Other *bbs* phenotypes, such as shortened cilia and defective DiI uptake (Figure 2E and data not shown), were not rescued by *gcy-35(lf)* mutants. These results suggest that the ciliary structures remain impaired in *gcy-35;bbs* mutants, and that the cellular pathways regulating body size, developmental timing and roaming behaviours either function genetically downstream of, or differ from those involved in sensation.

### The GCY-35/GCY-36 sGC complex influences the body size of *bbs* mutants through a subset of oxygen sensing body cavity neurons

GCY-35 and its partner GCY-36 form a heterodimeric sGC that modulates *C. elegans* behaviour in response to ambient oxygen concentrations [40]–[41]. *gcy-36(db66)* (*lf*) mutants [40] rescued the body size defect of *bbs-7* mutants (Figure 2B). Moreover, *gcy-35;gcy-36;bbs-7* animals exhibited a body size no different from either *gcy-35;bbs-7* or *gcy-36;bbs-7* (Figure 2B), consistent with GCY-35 and GCY-36 also functioning as a heterodimeric sGC to regulate body size.

**Figure 3:**
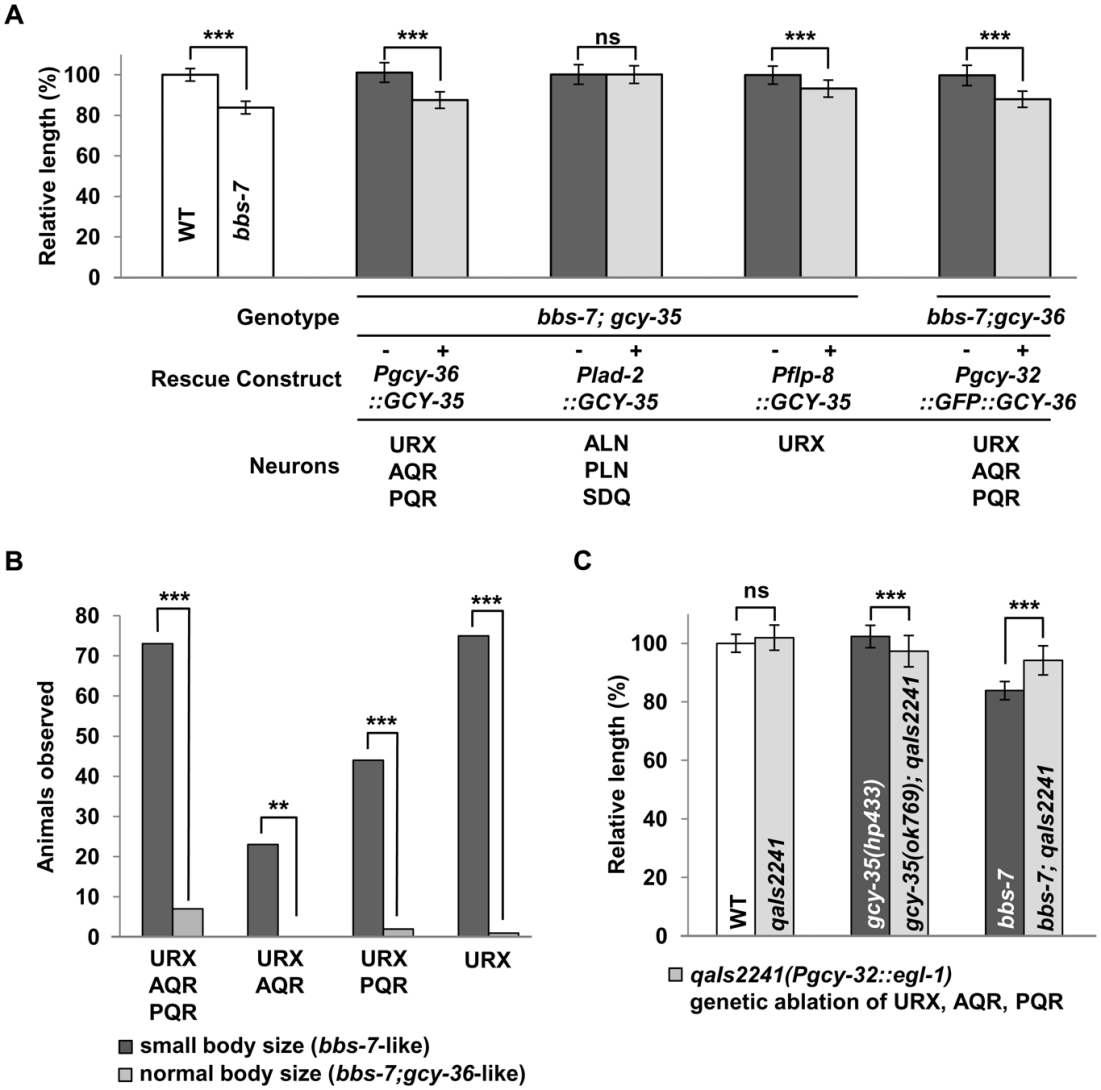
GCY-35 and GCY-36 are required in body cavity neurons to influence body size. (A) The body length of transgenic (+, light bars) *gcy-35;bbs-7* animals expressing GCY-35 or GFP::GCY-36 constructs versus non-transgenic siblings (−, dark bars). Endogenous GCY-35 expressed in the body cavity neurons (*Pgcy-32, Pgcy-36*: URX, AQR, PQR), reverted *gcy-35(lf)*-mediated body size suppression, while expression in other endogenous GCY-35-expressing neurons (*Plad-2*: ALN, PLN, SDQ) did not. Expression of GCY-35 in URX and non-overlapping neurons (*Pflp-8*: URX, ASE, PVM) partially reverted the suppression in transgenic *gcy-35;bbs-7* animals. ANOVA with Tukey, *** p<0.001, ns – p≥0.05, n≥20. Data represent mean ± SD normalized against wild-type body length. (B) Mosaic analysis of *bbs-7;gcy-36* transgenic animals expressing GFP::GCY-36 showed a requirement for URX expression in rescuing suppression effects. Animals were first separated by body size into two groups: small body size (*bbs-7-like*, dark bars) or normal body size (*bbs-7;gcy-36*-like, light bars); they were then examined for the presence or absence of GFP::GCY-36 expression in the AQR, PQR and URX neurons. Fisher's Exact test, *** p<0.001; ** p<0.01, n≥20. Y-axis indicates the pooled number of animals of respective neuronal expression patterns. (C) The genetic ablation of AQR, PQR, and URX neurons using the *qaIs2241 (pgcy-32::egl-1)* transgene rescued the body size defects of *bbs-7* mutants. The body length of wild-type, *gcy-35* and *bbs-7* animals (dark bars) was compared to those in combination with *qaIs2241* (light bars). ANOVA with Tukey, *** p<0.001; ns – p≥0.05, n≥20. Data represent mean ± SD normalized against wild-type body length. For *bbs-7* dataset, the *ok1351* deletion allele was used.

We examined in which neurons this sGC influences body size using *bbs* mutants. Both GCY-35 and GCY-36 are expressed in the ciliated body cavity sensory neurons AQR and PQR, and a non-ciliated body cavity neuron URX; GCY-35 is additionally expressed in the non-ciliated ALN, PLN, and SDQ neurons [41]. AQR, PQR and URX expression of wild-type GCY-35 in *gcy-35;bbs-7* animals reverted body size similarly to that of *bbs-7* mutants, whereas GCY-35 expression in ALN, PLN, and SDQ had no effect (Figure 3A). Similarly, the expression of a GFP-tagged GCY-36 in AQR, PQR and URX neurons in *bbs-7;gcy-36* animals was also sufficient to revert body size close to that of *bbs-7* mutants (Figure 3A). This suggests a specific requirement of GCY-35 and GCY-36 in body cavity neurons to regulate body size.

Among them, URX appears to be the most essential neuron, as restoring GCY-35 in URX (and other neurons not normally expressing *gcy-35/gcy-36*) by an exogenous promoter showed a partial, but significant reversion of the body size in *gcy-35;bbs-7* animals (Figure 3A). Similarly, GCY-36 is most essential in URX: we conducted a mosaic analysis of *bbs-7;gcy-36* animals carrying a functional GFP::GCY-36 transgene, and we observed expression of GFP::GCY-36 in URX of all rescued animals (Figure 3B). URX expression of the GCY-35/GCY-36 sGC is therefore essential, although not fully sufficient, to regulate the body size of *bbs* mutants.

Finally, if the body cavity neurons contribute to the small size phenotype of *bbs* mutants, their ablation by the transgene *qaIs2241*(*Pgcy-36::EGL-1*) [42] in a *bbs* background should also have a rescuing effect. While both *qaIs2241* and *gcy-35;qaIs2241* animals exhibited normal body sizes, *bbs-7;qaIs2241* mutants showed a significantly increased body size when compared to *bbs-7* animals (Figure 3C), further supporting that these neurons modulate the body size of *bbs* mutants.

### The expression and localization of GFP::GCY-35 and GFP::GCY-36 are grossly normal in *bbs* mutants

Our genetic analyses indicate that BBS proteins negatively regulate sGC-mediated signalling activity. We examined if *bbs* mutants exhibit an elevated expression and/or expanded localization of sGC in these neurons. As reported [40], a functional GFP::GCY-36 localized largely to the soma and along the dendrites of AQR, PQR and URX. We did not observe significant changes in its expression or localization in *bbs* mutants (Figure S3), nor did we see a loss of ciliary localization as reported for the loss of a putative isoprenylation signal at the C-terminus [40]. *bbs* mutations did not perturb, at a gross level, the expression and localization of GFP::GCY-35 either (Figure S3). We did observe a high degree of morphological variability in the dendritic endings of URX, AQR, and PQR neurons as previously reported [43] in both wild-type and *bbs* mutants. However, given the morphological variability, we cannot exclude the possibility of subtle alterations in the cilium length of *bbs* mutants. Therefore, while AQR, PQR and URX neurons regulate the body size through a process that involves sGC activity, it does not appear to result directly from altered sGC protein level or subcellular localization.

### The cGMP-dependent protein kinase (PKG) EGL-4 is a sGC effector in body size regulation

cGMP is a key secondary messenger (reviewed in [44]). In *C. elegans*, cGMP activates a heteromeric cGMP-gated ion channel TAX-2/TAX-4 for oxygen sensation and other sensory processes [45]. cGMP also activates a PKG, EGL-4, to regulate olfactory adaptation, life span, behavioural states and body size [33]–[34], [46]–[47]. Specifically, *egl-4(lf)* mutants exhibit a large body size, and are epistatic to the reduced body size and roaming behaviour of some sensory mutants; whereas a constitutively active, gain-of-function (*gf*) *egl-4* mutation, causes a small body size [36]. The shared effects of *bbs* and *egl-4* suggested that EGL-4 could be a downstream effector of sGCs in body size regulation.

**Figure 4:**
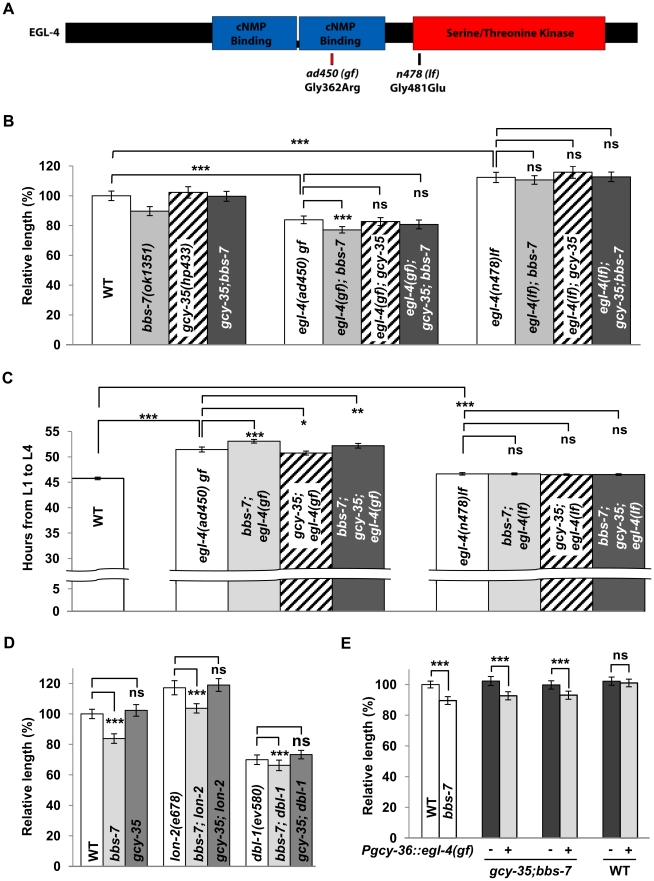
EGL-4 is an effector of GCY-35/GCY-36 in influencing body length and developmental timing. (A) A schematic of EGL-4 showing allelic information on the *ad450sd* (gain-of-function) and *n478* (loss-of-function). *egl-4(gf)* and *egl-4(lf)* are epistatic to *bbs-7(ok1351)* (gray bars), *gcy-35(hp443)* (diagonal striped bars) and *gcy-35;bbs-7* (dark bars) for both body size (B) and developmental timing (C). ANOVA with Tukey, *** p<0.001; ns – p≥0.05 or body length difference <3.5%. For body length, data represent mean ± SD normalized against wild-type body length, n≥20. For developmental timing, data represent mean (hours) ± SD, n≥50, N≥10 replicates. (D) Loss of function mutations in the TGF-β pathway (*BMP-5*/*dbl-1* and *lon-2*, white bars) had an additive effect on regulating body size in conjunction with *bbs* mutants (light bars), while being unaffected by the loss of *gcy-35(ok769)* (dark bars). ANOVA with Tukey, *** p<0.001; ns – p≥0.05, n≥20. Data represent mean ± SD normalized against wild-type body length. (E) The transgenic expression (+, light bars) of *egl-4(gf)* in body cavity neurons reverted the body size suppression in *gcy-35;bbs-7* in comparison to non-transgenic siblings (−, dark bars). EGL-4(gf) expression within body cavity neurons did not affect the body size of wild-type transgenic controls. ANOVA with Tukey, *** p<0.001; ns – p≥0.05, n≥20. Data represent mean ± SD normalized against wild-type body length.

We examined the *egl-4* mutants (Figure 4) for their genetic interactions with *bbs-7* and *gcy-35* mutants. *egl-4(lf)* alleles were epistatic to *bbs-7*, *gcy-35* and *bbs-7;gcy-35* for body size and developmental timing (Figure 4B and 4C). *egl-4(gf)* also exhibited an epistatic effect to *bbs-7* and *bbs-7;gcy-35* mutants, although *egl-4(gf);bbs-7* and *egl-4(gf);bbs-7;gcy-35* mutants may exhibit a slightly more severe phenotype than *egl-4(gf)* that was only distinguished by statistical analyses (Figure 4B). By contrast, *lf* mutants for another cGMP effector, the cGMP-gated cation channel subunit TAX-2 and TAX-4, did not exert body size suppression for *bbs-7* (Figure S4). Moreover, some TGF-β signalling mutants exhibited altered body size [48]–[50]. While EGL-4 was proposed to function genetically downstream of TGF-β signalling [33], body size defects of several TGF-β mutants exhibited additive effects in *bbs-7* or *bbs-7;gcy-35* backgrounds (Figure 4D), suggesting that TGF-β signalling and BBS-mediated body size regulation likely operates in an additive or parallel manner. Taken together, these results confirm a specific genetic relationship between *bbs-7*, *gcy-35* and *egl-4* for cilia-mediated body size regulation and developmental timing.

To further test if the body size influence of GCY-35/GCY-36 sGC functions through EGL-4, we expressed *egl-4* cDNA harbouring the *ad450(gf)* mutation in AQR, PQR and URX neurons of *gcy-35;bbs-7* animals. If GCY-35 regulates body size through activating EGL-4, a constitutively activated EGL-4(*ad450*) in these neurons should abrogate the body size suppression by the loss of *gcy-35*. Indeed, this transgene reverted animals to a *bbs-7*-like body size (Figure 4E). Wild-type animals expressing the same transgene did not exhibit changes in body size (Figure 4E). Not only is this result consistent with elevated cGMP signalling contributing to the reduced body size in *bbs* mutants, it further suggests that GCY-35/GCY-36 regulates body size through EGL-4 in the body cavity neurons.

### BBS proteins are required in multiple sensory neurons to regulate body size

**Figure 5:**
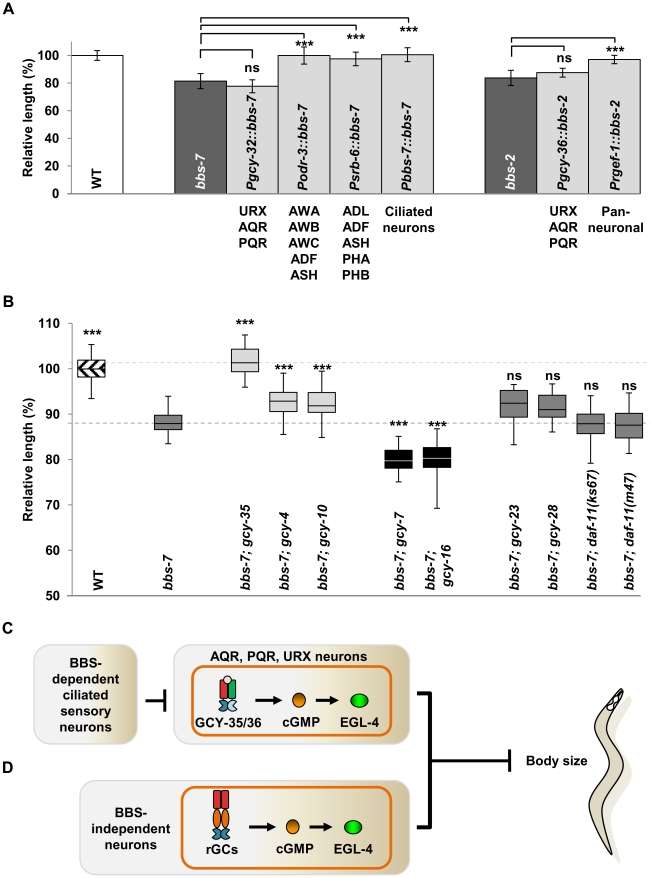
Multiple sensory neurons influence the body size of *bbs* mutants. (A) The expression of wild-type BBS protein in body cavity neurons was insufficient to rescue body length defects of *bbs-7* and *bbs-2* mutants, while expression in other ciliated sensory neurons (light bars) restored body length. ANOVA with Tukey, *** p<0.001; ns – p≥0.05, n≥11. Data represent mean ± SD normalized against wild-type body length. (B) GCY-35, but not other GCY mutants, exhibited a strong suppression effect on *bbs-7* mutant body length. Box and whisker plot of additional *gcy* mutants showing partially improved body length (light bars), decreased body length (black bars), and no change (dark bars) in comparison to *bbs-7* mutants. Boxes represent 25^th^–75^th^ percentile of populations with maximum and minimum values as whiskers. ANOVA with Tukey, *** p<0.001; ns – p≥0.05 compared to *bbs-7* mutants. Data represent mean ± SD normalized against wild-type body length. (C–D) A model for ciliary and cGMP-mediated body size regulation. (C) GCY-35/GCY-36 activity produces cGMP in the body cavity neurons and activates EGL-4 to inhibit body size. This process is influenced by BBS proteins present in other ciliated sensory neurons. (D) Some ciliated sensory neurons producing other GCs undergo a BBS-independent EGL-4 activation to influence body size and developmental timing.

To investigate whether ciliary functions regulate body size strictly through body cavity neurons like GCY-35/GCY-36, we expressed a functional BBS-7 or GFP::BBS-2 in the AQR, PQR and URX neurons of *bbs-7* or *bbs-2* mutants, respectively. The small body size of respective *bbs* mutants was not rescued (Figure 5A). We did, however, observe a complete rescue of the body size with a pan-neuronally expressed *GFP::BBS-2* in *bbs-2* mutants (Figure 5A). Moreover, we observed a full or partial rescue of the body size defects when a functional BBS-7 was expressed in at least two non-overlapping groups of sensory neurons AWB, AWC, AWA, ADF and ASH, or, ADL, ADF, ASH, PHA and PHB (Figure 5A). All these neurons have sensory cilia exposed to outside of the body cavity. Therefore, while the GCY-35/GCY-36 sGC functions through body cavity neurons to regulate body size, restoring ciliary function in these neurons alone does not sufficiently reduce cGMP signalling to restore body size. Alternatively, other sensory neurons that input onto the body cavity neurons, may serve to regulate body size. Regardless, the observation that BBS proteins can influence body size through different groups of sensory neurons is reminiscent of the previously reported observation that the body size of *egl-4(lf)* mutants could be rescued by restoring EGL-4 expression in non-overlapping sets of sensory neurons [33].

### GCY-35/GCY-36 acts as a predominant effector in cilia-mediated body size regulation

That multiple, non-overlapping and non-body cavity ciliated sensory neurons (CSNs) regulate body size suggests a cumulative effect on cGMP signalling-mediated body size regulation by multiple sensory neurons, or, a predominating effect by body cavity neurons in cilia-mediated body size regulation through GCY-35/GCY-36. The first model predicts that additional GCs in other sensory neurons would epistatically suppress body size. The *C. elegans* genome encodes 7 sGCs and 27 receptor-like GCs (rGCs) [51]. Single loss-of-function mutations in other sGCs, GCY-31, 32, 33, 34 and 37 all failed to alter *bbs-7* body size defects (Figure S5A and data not shown). Of 16 rGC mutants tested, four exhibited modifying effects, two very mildly suppressing and two significantly exacerbating the smaller body size of *bbs-7* animals, but none suppressed *bbs-7* mutant phenotypes comparably to *gcy-35/gcy-36* (Figure 5B and Figure S5A). We examined the effect of several double and triple rGC mutants on *bbs-7* body size to explore the possibility that multiple rGCs function redundantly [52] but we did not observe obvious suppression effect in these additional mutants (Figure S5A).

We further examined the combinational effect of *gcy-35* and other rGC mutants on *bbs-7*, including a mildly suppressing allele (*gcy-4(tm1653*)), two exacerbating alleles (*gcy-7(tm901)* and *gcy-16(ok2538)*) and three “neutral” alleles (*gcy-23(ok797)*, *gcy-28(tm2411)* and *gcy-25(tm4300)*). We did not observe a significant body size improvement between *gcy-35;bbs-7* and these triple mutants (Figure S5B). Therefore with the caveat that we have not exhausted the examination of all single or combinational GC mutants, GCY-35/GCY-36, through the body cavity neurons, act uniquely as a predominating effector for BBS-mediated body size regulation.

## Discussion

In the present study, we show that *C. elegans bbs* mutants exhibit reduced body length, delayed development and altered roaming pattern, in addition to known sensory defects. These endophenotypes depend, fully or in part, on the GCY-35/GCY-36 sGC complex, through its effector EGL-4 PKG, in the AQR, PQR and URX body cavity neurons. On the other hand, body size can also be regulated via multiple, non-overlapping sets of non-body cavity sensory neurons. We propose that the loss of *C. elegans* BBS function in ciliated sensory neurons leads to non-cell autonomous, aberrant cGMP-PKG signalling in body cavity neurons, which contributes to abnormal body size and delayed development.

### *C. elegans bbs* mutants exhibit non-cell autonomous endophenotypes

Ciliated sensory neurons transduce environmental cues into behavioural responses. In *C. elegans bbs* mutants, defective IFT and ciliary functions are reflected by chemosensory and thermosensory deficits [13]–[14]. Given the restricted expression of *C. elegans* BBS proteins in sensory neurons, the additional *bbs* endophenotypes such as developmental timing, body and inferred cell size, and roaming indicate that in addition to sensory perception, sensory neurons also participate in developmental regulation in a non-cell autonomous manner. These *bbs* endophenoptypes are not recapitulated by several *dyf*/IFT motor mutants, further implying that BBS proteins affect sensory neuron function in addition to their role in IFT.

While all *bbs* mutants share these endophenotypes, they exhibit small differences in the severity of phenotypic expression that could be attributed to specific allelic effects. Alternatively, BBS proteins could possess certain degrees of unknown functional specificity. This may not be so surprising given the difference in phenotypic expression among BBS patient populations [53]–[55], as well as the observation that tissue-specific BBS isoforms are responsible for some syndromic features [22], [56].

The involvement of primary cilia in signalling during development [57] also positions them to affect development in a non-autonomous fashion. For example, mouse BBS proteins are required in the hypothalamus to regulate leptin receptor trafficking and to prevent the onset of obesity [31]. Ciliary dysfunction therefore contributes to increased adiposity partly in a non-cell autonomous manner. The additional phenotypes of *C. elegans bbs* mutants, highlights the global and non-cell autonomous consequence of sensory ciliary dysfunction, which may also account for some phenotypic features in other ciliopathy models.

### The GCY-35/GCY-36 sGC regulates body size through a mechanism divergent from oxygen sensing

Previous studies established that the GCY-35/GCY-36 sGC can regulate oxygen sensation through either the body cavity neurons, or another group of neurons [40]–[42]. Activated by oxygen, this complex catalyzes the conversion of GTP to cGMP, which subsequently activates the cGMP-gated cation channel TAX-2/TAX-4 to initiate hyperoxic avoidance responses [45]. Additional sGCs can act in body cavity neurons or other neurons under specific hypoxic conditions [45], [58].

GCY-35/GCY-36 modifies body size through a mechanism partly divergent from that of hyperoxic avoidance. GCY-35 is only necessary and sufficient in body cavity neurons that either have ciliated dendrites [59] or express some ciliated neuron-specific genes [60]. Furthermore, the loss of EGL-4, but not TAX-2 or TAX-4, suppresses the body size defects of *bbs* and *dyf* mutants. The loss of TAX-2 and TAX-4, in fact, slightly exacerbated *bbs* phenotypes (Figure S4), which may reflect an increased cGMP pool for EGL-4 activation or the loss of a potential EGL-4 phosphorylation target [35]. As well, despite some sGCs having overlapping expression profiles with GCY-35/GCY-36, other oxygen-responsive sGC mutants failed to suppress *bbs-7* body size defects under standard culture conditions - possibly due to low activity under normoxia. Therefore, body cavity neurons, through GCY-35/GCY-36 activity, participate in developmental regulation through an alternate cGMP effector.

EGL-4 is present fairly ubiquitously, but the activation of EGL-4 in sensory neurons exerts a dominant influence on body size [33]. The genetic epistasis of both *egl-4(lf)* and *egl-4(gf)* alleles over that of *bbs* and *bbs;gcy-35* argues in favour of BBS proteins and EGL-4 functioning through a shared cellular pathway to regulate body size and developmental timing. Expression of EGL-4(*gf*) in the body cavity neurons of *gcy-35;bbs-7* mutants specifically alleviated the rescuing effect on body size, suggesting that increased EGL-4 activity, driven by increased availability of cGMP in body cavity neurons, contributes to the body size defects of some ciliary mutants (Figure 5C).

### BBS proteins affect body size indirectly through body cavity neurons

The body size defects of ciliary mutants are rescued by non-overlapping sets of sensory neurons. However, restoring BBS function in body cavity neurons is insufficient to rescue the observed body size defects, giving rise to a possibility that the effect of cGMP signalling by body cavity neurons is indirectly moderated by a non-cell autonomous function of BBS proteins in ciliated sensory neurons. Furthermore, that URX, a pair of non-ciliated neurons, play a necessary role in this suppression indicates that BBS proteins are not directly influencing body size in these neurons. Our genetic analyses of the modifying effect of other GC mutants also support this scenario, as we have not found additional GC mutants that potently restore the body size of *bbs* mutants.

These results do not exclude the possibility that other GCs function redundantly in non-body cavity sensory neurons to influence body size through EGL-4/PKG (Figure 5D). The overexpression of *egl-4(gf)* in body cavity neurons was incapable of further reducing the body size of *gcy-35;bbs-7* animals beyond that of *bbs-7* mutants. This is in concordance with the ablation of body cavity neurons, which did not phenocopy the large body size of EGL-4 loss of function mutants, suggesting additional neuronal groups influence body size through EGL-4/PKG signalling. This study, however, establishes body cavity neurons as a predominating cGMP/PKG effector in body size regulation, and the ciliated sensory neurons as playing a key role in moderating cGMP signalling of these effector neurons.

Mechanisms on how dysfunctional ciliary sensory neurons lead to elevated cGMP/PKG signalling in these neurons are unknown. The body cavity neurons, AQR, PQR and URX do not receive extensive or direct synaptic inputs from sensory neurons where BBS proteins are sufficient to rescue body size. The non-cell autonomous effect of ciliated sensory neurons therefore suggests a potential involvement of indirect synaptic inputs, or other forms of neuronal communications, such as peptidergic and/or hormonal signalling between these neuronal groups. For example, body cavity neurons express the *C. elegans* homologue of the neuropeptide NPY receptor [61], making their activity susceptible to modulation by neuropeptides, some of which could be secreted by sensory neurons [62]. Sensory neurons also secrete insulin/IGF-like ligands, some of which may systematically affect neuronal states [63]–[64]. Indeed, insulin and leptin have been shown to regulate the activity of specific hypothalamic neurons [65]–[66]. Speculatively, *C. elegans* BBS proteins could affect the secretion of multiple signals by ciliated sensory neurons to regulate cGMP/EGL-4 signalling in the body cavity neurons.

### A potential involvement of aberrant cGMP signalling in ciliopathies

While aberrant PCP, Shh and Wnt signalling underlie a number of ciliopathy features, the biology behind other ciliopathy features such as photoreceptor degeneration, and reduced body size in *Bbs* mice [15] remains unexplained. cGMP signalling plays key roles in biological processes such as phototransduction, axonal guidance, and synaptic plasticity (reviewed in [67]–[68]). PKGs have also been implicated in photoreceptor degeneration and dwarfism [69]–[70]. It is worth exploring the involvement of cGMP signalling in the underlying pathology of BBS and other ciliopathy features.

## Materials and Methods

### Strains

All strains were maintained on NGM plates at 20°C. *C. elegans bbs*, *gcy* and *egl-4* strains were obtained from the *CGC*. CX7102 was obtained from the Bargmann lab. Genotypes for all strains are listed in Text S1.

### Mapping and cloning of *hp433*

*bbs-7(n1606);hp433* mutants were outcrossed twice against N2 by selecting animals that were genotyped for *n1606* mutation, but exhibited normal body size. The *hp433* mutation was crossed into *bbs-7(ok1351)* mutants and mapped based on the suppression of small body size and roaming defects using the SNP markers in the CB4856 strain, which placed it at a 93.5 kb interval between the SNPs *pkp1133* and *uCE1-1426*. We conclude that *hp433* encodes *gcy-35* by: 1) Injection of three overlapping fosmids covering *gcy-35*, T04D3.5, and T04D3.t2, reverted the body size suppression in *hp433; bbs-7* animals. A fragment of WRM641cB09 that encompassed a truncated *gcy-35*, but complete T04D3.5 and T04D3.t2 failed to revert the *hp433* suppression; A genomic fragment containing only *gcy-35* fully reverted the suppression. 2) *gcy-35(ok769)* animals shared the same synthetic phenotypes and genetic interactions with *bbs-7* as *hp433*, while *hp433;bbs-7(ok1351)* animals also failed to complement *gcy-35(ok769);bbs-7(ok1351)*. 3) Sequencing of *gcy-35* identified a 2 bp deletion in exon 8.

### Molecular biology, *C. elegans* phenotype examination

See Text S1.

## Supporting Information

Figure S1 *bbs* mutants exhibit a smaller body size in late larvae and adulthood stages. The body length measurements of *bbs* mutants at L4 (A) and 66-hours post-L4(B) showed consistent size defects when compared to similarly staged wild-type animals. ANOVA with Tukey, *** p<0.001 in comparison to wild-type animals, n≥20. Data represent mean ± SD normalized against wild-type body length.
(TIF)

Figure S2 *dyf* (dye-filling) mutants show variable body size and genetic interaction with *gcy-35(lf)*. (A) The relative length of *dyf* mutants in comparison to wild-type animals. Some *dyf* mutants had variable degrees of body length defects, while others showed little to no change in body length. ANOVA with Tukey, *** p<0.001; ns – p≥0.05 or length difference <3.5% relative to wild-type animals, n≥30. Data represent mean ± SD normalized against wild-type body length. (B) The body size of only a subset of *dyf* mutants (dark boxes) was mildly altered by the loss of *gcy-35* (light boxes). Boxes represent 25^th^–75^th^ percentile of populations with maximum and minimum values as whiskers. ANOVA with Tukey, *** p<0.001; * p<0.05; ns – p≥0.05 or length difference <3.5%, n≥30. Data represent mean ± SD normalized against wild-type body length.
(TIF)

Figure S3 *bbs* mutants show no visible defects in GCY-35 or GCY-36 localisation. GFP signals by *Pgcy-36::GFP::GCY-35* or *Pgcy-32::GFP::GCY-36* expressed in *gcy-35(hp433)* (A–C) or *gcy-36(db66)* (G–I) mutants. Strong signals were observed in the soma (orange arrowheads) and tips of the dendrites (white arrowheads) in AQR, PQR, and in the soma and dendrites of URX neurons. Expression of the same constructs in a *gcy-35;bbs-7* (D–F) or *bbs-7;gcy-36* (J–L) backgrounds exhibited no gross changes to localization in comparison to wild-type animals. Shown here are representative images of young adult animals.
(TIF)

Figure S4 The cGMP-gated ion channel TAX-2/4 does not suppress the body size defects of *bbs* mutants (A) *tax-2* and *tax-4* failed to rescue *bbs-7* mutant body size defects. ANOVA with Tukey, *** p<0.001; ns – p≥0.05 or length difference <3.5%, n≥20. Data represent mean ± SD normalized against wild-type body length. (B) Developmental timing in *tax-2;bbs-7* mutants was further delayed in comparison to *bbs-7* mutant populations. ANOVA, *** p<0.001, n≥50, N≥10 replicates. Data represent mean (hours) ± SD. (C) Roaming defects of *tax-2;bbs-7* and *tax-4 bbs-7* animals were no different compared to *bbs-7* single animals. Kruskal-Wallis with Dunn's, ns – p≥0.05, n≥25, N≥2 replicates. Data represent mean squares roamed ± SEM.
(TIF)

Figure S5 Mutations in other guanylate cyclases do not suppress *bbs-7* body size defects. (A) Mutant alleles of multiple sGC genes (gray bars) and rGC genes (dark bars) did not exhibit a significant modifying effect on *bbs-7* mutants. The loss of functionally redundant rGCs (*gcy-8, -18, -23*) [52] does not significantly modify *bbs-7* body size defects either (black bars). ANOVA with Tukey, ns – p<0.05, n≥30. (B) Suppression of *bbs-7* body size by the loss of *gcy-35* was not significantly influenced by the loss of additional rGCs. ANOVA with Tukey, *** p<0.001; ** p<0.01; ns – p≥0.05, n≥20. All data represent mean ± SD normalized against wild-type body length.
(TIF)

Table S1 A list of the *bbs* mutant alleles used in this study, outlining the type of change or deletion characterized in these strains and the resulting changes to protein translation or domains.
(DOC)

Text S1 Text S1 includes a list of strains generated and used in this study, and methods for molecular biology, phenotype analyses, statistical analyses and fluorescent microscopy.
(DOC)

## References

[1] Berbari NF, O'Connor AK, Haycraft CJ, Yoder BK (2009). The primary cilium as a complex signaling center.. Curr Biol.

[2] Tobin JL, Beales PL (2009). The nonmotile ciliopathies.. Genet Med.

[3] Baker K, Beales PL (2009). Making sense of cilia in disease: the human ciliopathies.. Am J Med Genet C Semin Med Genet.

[4] Kim SK, Shindo A, Park TJ, Oh EC, Ghosh S (2010). Planar cell polarity acts through septins to control collective cell movement and ciliogenesis.. Science.

[5] Otto EA, Hurd TW, Airik R, Chaki M, Zhou W (2010). Candidate exome capture identifies mutation of SDCCAG8 as the cause of a retinal-renal ciliopathy.. Nat Genet.

[6] Nachury MV, Loktev AV, Zhang Q, Westlake CJ, Peranen J (2007). A core complex of BBS proteins cooperates with the GTPase Rab8 to promote ciliary membrane biogenesis.. Cell.

[7] Jin H, White SR, Shida T, Schulz S, Aguiar M (2010). The conserved Bardet-Biedl syndrome proteins assemble a coat that traffics membrane proteins to cilia.. Cell.

[8] Ou G, Blacque OE, Snow JJ, Leroux MR, Scholey JM (2005). Functional coordination of intraflagellar transport motors.. Nature.

[9] Pan X, Ou G, Civelekoglu-Scholey G, Blacque OE, Endres NF (2006). Mechanism of transport of IFT particles in C. elegans cilia by the concerted action of kinesin-II and OSM-3 motors.. J Cell Biol.

[10] Zaghloul NA, Katsanis N (2009). Mechanistic insights into Bardet-Biedl syndrome, a model ciliopathy.. J Clin Invest.

[11] Inglis PN, Ou G, Leroux MR, Scholey JM (2007). The sensory cilia of Caenorhabditis elegans.. WormBook.

[12] Ansley SJ, Badano JL, Blacque OE, Hill J, Hoskins BE (2003). Basal body dysfunction is a likely cause of pleiotropic Bardet-Biedl syndrome.. Nature.

[13] Blacque OE, Reardon MJ, Li C, McCarthy J, Mahjoub MR (2004). Loss of C. elegans BBS-7 and BBS-8 protein function results in cilia defects and compromised intraflagellar transport.. Genes Dev.

[14] Tan PL, Barr T, Inglis PN, Mitsuma N, Huang SM (2007). Loss of Bardet Biedl syndrome proteins causes defects in peripheral sensory innervation and function.. Proc Natl Acad Sci U S A.

[15] Nishimura DY, Fath M, Mullins RF, Searby C, Andrews M (2004). Bbs2-null mice have neurosensory deficits, a defect in social dominance, and retinopathy associated with mislocalization of rhodopsin.. Proc Natl Acad Sci U S A.

[16] Eichers ER, Abd-El-Barr MM, Paylor R, Lewis RA, Bi W (2006). Phenotypic characterization of Bbs4 null mice reveals age-dependent penetrance and variable expressivity.. Hum Genet.

[17] Ross AJ, May-Simera H, Eichers ER, Kai M, Hill J (2005). Disruption of Bardet-Biedl syndrome ciliary proteins perturbs planar cell polarity in vertebrates.. Nat Genet.

[18] Kulaga HM, Leitch CC, Eichers ER, Badano JL, Lesemann A (2004). Loss of BBS proteins causes anosmia in humans and defects in olfactory cilia structure and function in the mouse.. Nat Genet.

[19] Badano JL, Leitch CC, Ansley SJ, May-Simera H, Lawson S (2006). Dissection of epistasis in oligogenic Bardet-Biedl syndrome.. Nature.

[20] Yen HJ, Tayeh MK, Mullins RF, Stone EM, Sheffield VC (2006). Bardet-Biedl syndrome genes are important in retrograde intracellular trafficking and Kupffer's vesicle cilia function.. Hum Mol Genet.

[21] Tayeh MK, Yen HJ, Beck JS, Searby CC, Westfall TA (2008). Genetic interaction between Bardet-Biedl syndrome genes and implications for limb patterning.. Hum Mol Genet.

[22] Pretorius PR, Baye LM, Nishimura DY, Searby CC, Bugge K (2010). Identification and functional analysis of the vision-specific BBS3 (ARL6) long isoform.. PLoS Genet.

[23] Corbit KC, Aanstad P, Singla V, Norman AR, Stainier DY (2005). Vertebrate Smoothened functions at the primary cilium.. Nature.

[24] Rohatgi R, Milenkovic L, Scott MP (2007). Patched1 regulates hedgehog signaling at the primary cilium.. Science.

[25] Huangfu D, Liu A, Rakeman AS, Murcia NS, Niswander L (2003). Hedgehog signalling in the mouse requires intraflagellar transport proteins.. Nature.

[26] Jones C, Roper VC, Foucher I, Qian D, Banizs B (2008). Ciliary proteins link basal body polarization to planar cell polarity regulation.. Nat Genet.

[27] Corbit KC, Shyer AE, Dowdle WE, Gaulden J, Singla V (2008). Kif3a constrains β-catenin-dependent Wnt signalling through dual ciliary and non-ciliary mechanisms.. Nat Cell Biol.

[28] Lin F, Hiesberger T, Cordes K, Sinclair AM, Goldstein LS (2003). Kidney-specific inactivation of the KIF3A subunit of kinesin-II inhibits renal ciliogenesis and produces polycystic kidney disease.. Proc Natl Acad Sci U S A.

[29] Schneider L, Clement CA, Teilmann SC, Pazour GJ, Hoffmann EK (2005). PDGFRalphaalpha signaling is regulated through the primary cilium in fibroblasts.. Curr Biol.

[30] Berbari NF, Johnson AD, Lewis JS, Askwith CC, Mykytyn K (2008). Identification of ciliary localization sequences within the third intracellular loop of G protein-coupled receptors.. Mol Biol Cell.

[31] Seo S, Guo DF, Bugge K, Morgan DA, Rahmouni K (2009). Requirement of Bardet-Biedl syndrome proteins for leptin receptor signaling.. Hum Mol Genet.

[32] Marion V, Stoetzel C, Schlicht D, Messaddeq N, Koch M (2009). Transient ciliogenesis involving Bardet-Biedl syndrome proteins is a fundamental characteristic of adipogenic differentiation.. Proc Natl Acad Sci U S A.

[33] Fujiwara M, Sengupta P, McIntire SL (2002). Regulation of body size and behavioral state of C. elegans by sensory perception and the EGL-4 cGMP-dependent protein kinase.. Neuron.

[34] Hirose T, Nakano Y, Nagamatsu Y, Misumi T, Ohta H (2003). Cyclic GMP-dependent protein kinase EGL-4 controls body size and lifespan in C elegans.. Development.

[35] L'Etoile ND, Coburn CM, Eastham J, Kistler A, Gallegos G (2002). The cyclic GMP-dependent protein kinase EGL-4 regulates olfactory adaptation in C. elegans.. Neuron.

[36] Raizen DM, Cullison KM, Pack AI, Sundaram MV (2006). A novel gain-of-function mutant of the cyclic GMP-dependent protein kinase egl-4 affects multiple physiological processes in Caenorhabditis elegans.. Genetics.

[37] Ou G, Koga M, Blacque OE, Murayama T, Ohshima Y (2007). Sensory ciliogenesis in Caenorhabditis elegans: assignment of IFT components into distinct modules based on transport and phenotypic profiles.. Mol Biol Cell.

[38] Brenner S (1973). The genetics of behaviour.. Br Med Bull.

[39] Koesling D, Friebe A (1999). Soluble guanylyl cyclase: structure and regulation.. Rev Physiol Biochem Pharmacol.

[40] Cheung BH, Arellano-Carbajal F, Rybicki I, de Bono M (2004). Soluble guanylate cyclases act in neurons exposed to the body fluid to promote C. elegans aggregation behavior.. Curr Biol.

[41] Gray JM, Karow DS, Lu H, Chang AJ, Chang JS (2004). Oxygen sensation and social feeding mediated by a C. elegans guanylate cyclase homologue.. Nature.

[42] Chang AJ, Chronis N, Karow DS, Marletta MA, Bargmann CI (2006). A distributed chemosensory circuit for oxygen preference in C. elegans.. PLoS Biol.

[43] Swoboda P, Adler HT, Thomas JH (2000). The RFX-type transcription factor DAF-19 regulates sensory neuron cilium formation in C. elegans.. Mol Cell.

[44] Lucas KA, Pitari GM, Kazerounian S, Ruiz-Stewart I, Park J (2000). Guanylyl cyclases and signaling by cyclic GMP.. Pharmacol Rev.

[45] Zimmer M, Gray JM, Pokala N, Chang AJ, Karow DS (2009). Neurons detect increases and decreases in oxygen levels using distinct guanylate cyclases.. Neuron.

[46] Nagamatsu Y, Ohshima Y (2004). Mechanisms for the control of body size by a G-kinase and a downstream TGF-β signal pathway in Caenorhabditis elegans.. Genes Cells.

[47] You YJ, Kim J, Raizen DM, Avery L (2008). Insulin, cGMP, and TGF-β signals regulate food intake and quiescence in C. elegans: a model for satiety.. Cell Metab.

[48] Gumienny TL, MacNeil LT, Wang H, de Bono M, Wrana JL (2007). Glypican LON-2 is a conserved negative regulator of BMP-like signaling in Caenorhabditis elegans.. Curr Biol.

[49] Morita K, Flemming AJ, Sugihara Y, Mochii M, Suzuki Y (2002). A Caenorhabditis elegans TGF-β, DBL-1, controls the expression of LON-1, a PR-related protein, that regulates polyploidization and body length.. EMBO J.

[50] Patterson GI, Padgett RW (2000). TGF-β-related pathways. Roles in Caenorhabditis elegans development.. Trends Genet.

[51] Ortiz CO, Etchberger JF, Posy SL, Frokjaer-Jensen C, Lockery S (2006). Searching for neuronal left/right asymmetry: genomewide analysis of nematode receptor-type guanylyl cyclases.. Genetics.

[52] Inada H, Ito H, Satterlee J, Sengupta P, Matsumoto K (2006). Identification of guanylyl cyclases that function in thermosensory neurons of Caenorhabditis elegans.. Genetics.

[53] Carmi R, Elbedour K, Stone EM, Sheffield VC (1995). Phenotypic differences among patients with Bardet-Biedl syndrome linked to three different chromosome loci.. Am J Med Genet.

[54] Beales PL, Warner AM, Hitman GA, Thakker R, Flinter FA (1997). Bardet-Biedl syndrome: a molecular and phenotypic study of 18 families.. J Med Genet.

[55] Billingsley G, Bin J, Fieggen KJ, Duncan JL, Gerth C (2010). Mutations in chaperonin-like BBS genes are a major contributor to disease development in a multiethnic Bardet-Biedl syndrome patient population.. J Med Genet.

[56] Pretorius PR, Aldahmesh MA, Alkuraya FS, Sheffield VC, Slusarski DC (2011). Functional analysis of BBS3 A89V that results in non-syndromic retinal degeneration.. Hum Mol Genet.

[57] Badano JL, Mitsuma N, Beales PL, Katsanis N (2006). The ciliopathies: an emerging class of human genetic disorders.. Annu Rev Genomics Hum Genet.

[58] Cheung BH, Cohen M, Rogers C, Albayram O, de Bono M (2005). Experience-dependent modulation of C. elegans behavior by ambient oxygen.. Curr Biol.

[59] Coates JC, de Bono M (2002). Antagonistic pathways in neurons exposed to body fluid regulate social feeding in Caenorhabditis elegans.. Nature.

[60] Kunitomo H, Uesugi H, Kohara Y, Iino Y (2005). Identification of ciliated sensory neuron-expressed genes in Caenorhabditis elegans using targeted pull-down of poly(A) tails.. Genome Biol.

[61] de Bono M, Bargmann CI (1998). Natural variation in a neuropeptide Y receptor homolog modifies social behavior and food response in C. elegans.. Cell.

[62] Rogers C, Reale V, Kim K, Chatwin H, Li C (2003). Inhibition of Caenorhabditis elegans social feeding by FMRFamide-related peptide activation of NPR-1.. Nat Neurosci.

[63] Pierce SB, Costa M, Wisotzkey R, Devadhar S, Homburger SA (2001). Regulation of DAF-2 receptor signaling by human insulin and ins-1, a member of the unusually large and diverse C. elegans insulin gene family.. Genes Dev.

[64] Cornils A, Gloeck M, Chen Z, Zhang Y, Alcedo J (2011). Specific insulin-like peptides encode sensory information to regulate distinct developmental processes.. Development.

[65] Belgardt BF, Husch A, Rother E, Ernst MB, Wunderlich FT (2008). PDK1 deficiency in POMC-expressing cells reveals FOXO1-dependent and -independent pathways in control of energy homeostasis and stress response.. Cell Metab.

[66] Klockener T, Hess S, Belgardt BF, Paeger L, Verhagen LA (2011). High-fat feeding promotes obesity via insulin receptor/PI3K-dependent inhibition of SF-1 VMH neurons.. Nat Neurosci.

[67] Schlossmann J, Feil R, Hofmann F (2005). Insights into cGMP signalling derived from cGMP kinase knockout mice.. Front Biosci.

[68] Hofmann F, Feil R, Kleppisch T, Schlossmann J (2006). Function of cGMP-dependent protein kinases as revealed by gene deletion.. Physiol Rev.

[69] Pfeifer A, Aszodi A, Seidler U, Ruth P, Hofmann F (1996). Intestinal secretory defects and dwarfism in mice lacking cGMP-dependent protein kinase II.. Science.

[70] Paquet-Durand F, Hauck SM, van Veen T, Ueffing M, Ekstrom P (2009). PKG activity causes photoreceptor cell death in two retinitis pigmentosa models.. J Neurochem.

