# Supplementary figures and images for: Mutations in a Guanylate Cyclase GCY-35/GCY-36 Modify Bardet-Biedl Syndrome–Associated Phenotypes in Caenorhabditis elegans
Source: PLoS Genet. 2011 Oct 13;7(10):e1002335. doi: 10.1371/journal.pgen.1002335 (PMC3192831; doi:10.1371/journal.pgen.1002335)

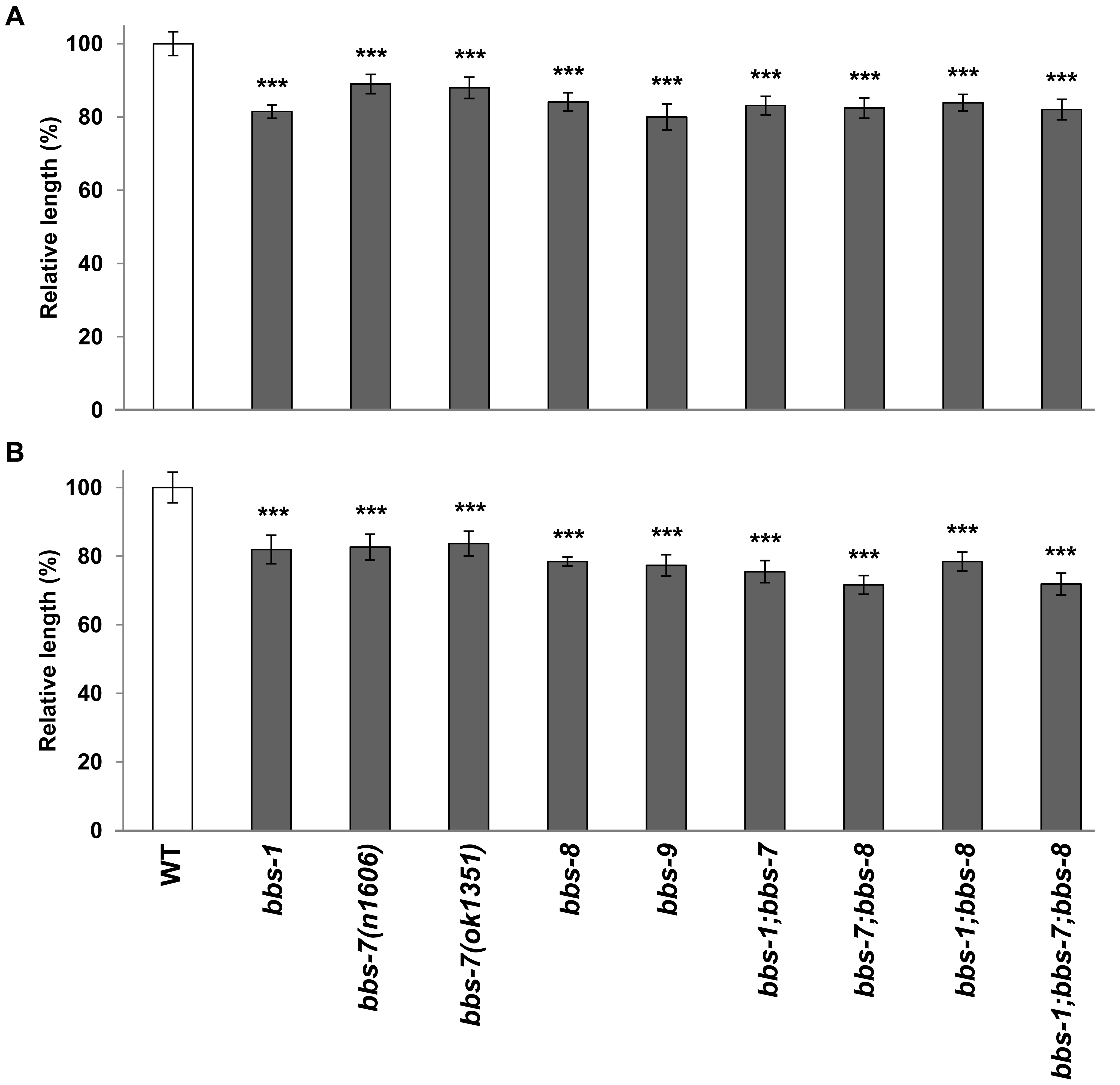

Supplement: Figure S1 — bbs mutants exhibit a smaller body size in late larvae and adulthood stages. The body length measurements of bbs mutants at L4 (A) and 66-hours post-L4(B) showed consistent size defects when compared to similarly staged wild-type animals. ANOVA with Tukey, *** p<0.001 in comparison to wild-type animals, n≥20. Data represent mean ± SD normalized against wild-type body length. (TIF) [file pgen.1002335.s001.tif]

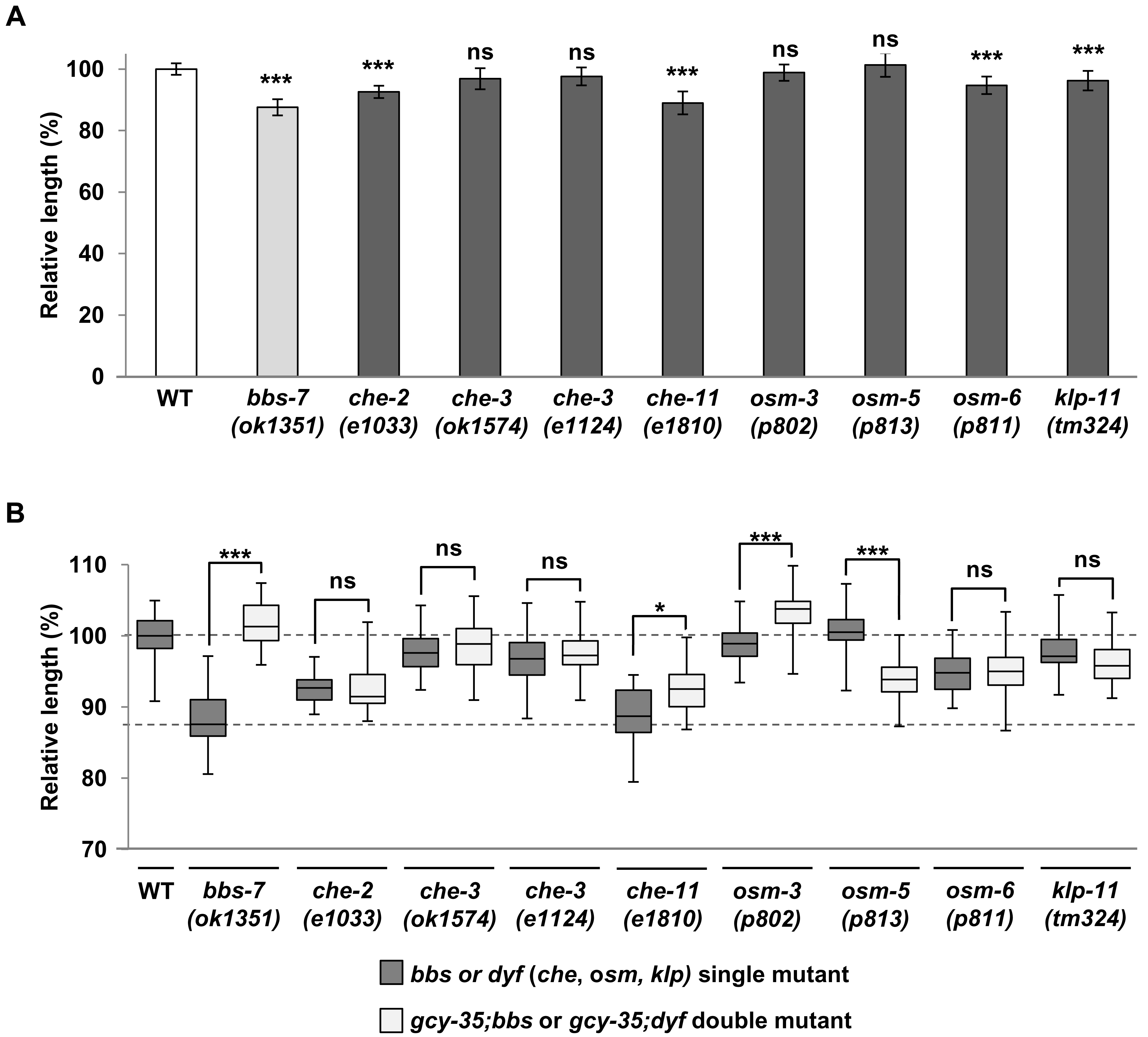

Supplement: Figure S2 — dyf (dye-filling) mutants show variable body size and genetic interaction with gcy-35(lf). (A) The relative length of dyf mutants in comparison to wild-type animals. Some dyf mutants had variable degrees of body length defects, while others showed little to no change in body length. ANOVA with Tukey, *** p<0.001; ns – p≥0.05 or length difference <3.5% relative to wild-type animals, n≥30. Data represent mean ± SD normalized against wild-type body length. (B) The body size of only a subset of dyf mutants (dark boxes) was mildly altered by the loss of gcy-35 (light boxes). Boxes represent 25th–75th percentile of populations with maximum and minimum values as whiskers. ANOVA with Tukey, *** p<0.001; * p<0.05; ns – p≥0.05 or length difference <3.5%, n≥30. Data represent mean ± SD normalized against wild-type body length. (TIF) [file pgen.1002335.s002.tif]

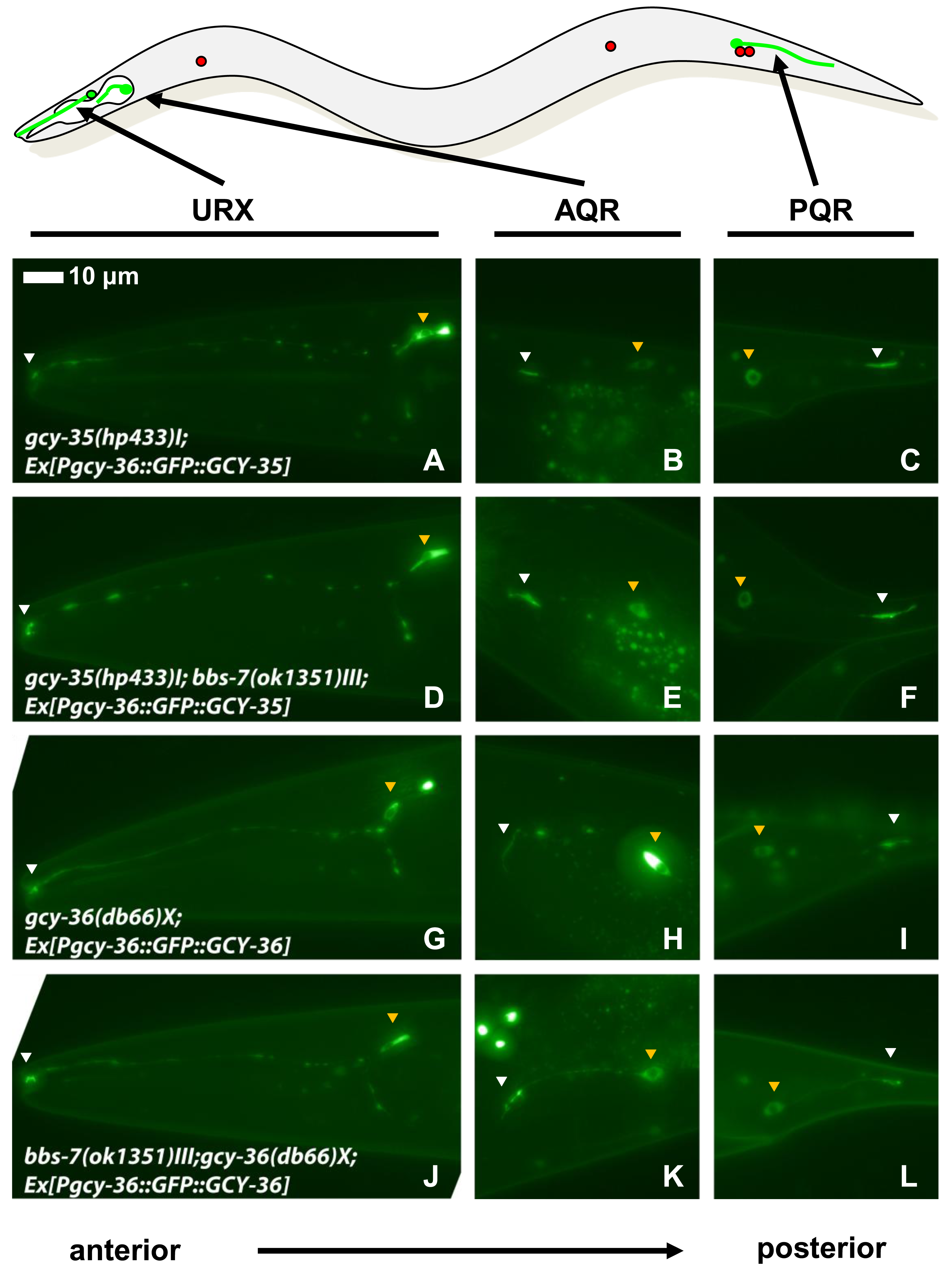

Supplement: Figure S3 — bbs mutants show no visible defects in GCY-35 or GCY-36 localisation. GFP signals by Pgcy-36::GFP::GCY-35 or Pgcy-32::GFP::GCY-36 expressed in gcy-35(hp433) (A–C) or gcy-36(db66) (G–I) mutants. Strong signals were observed in the soma (orange arrowheads) and tips of the dendrites (white arrowheads) in AQR, PQR, and in the soma and dendrites of URX neurons. Expression of the same constructs in a gcy-35;bbs-7 (D–F) or bbs-7;gcy-36 (J–L) backgrounds exhibited no gross changes to localization in comparison to wild-type animals. Shown here are representative images of young adult animals. (TIF) [file pgen.1002335.s003.tif]

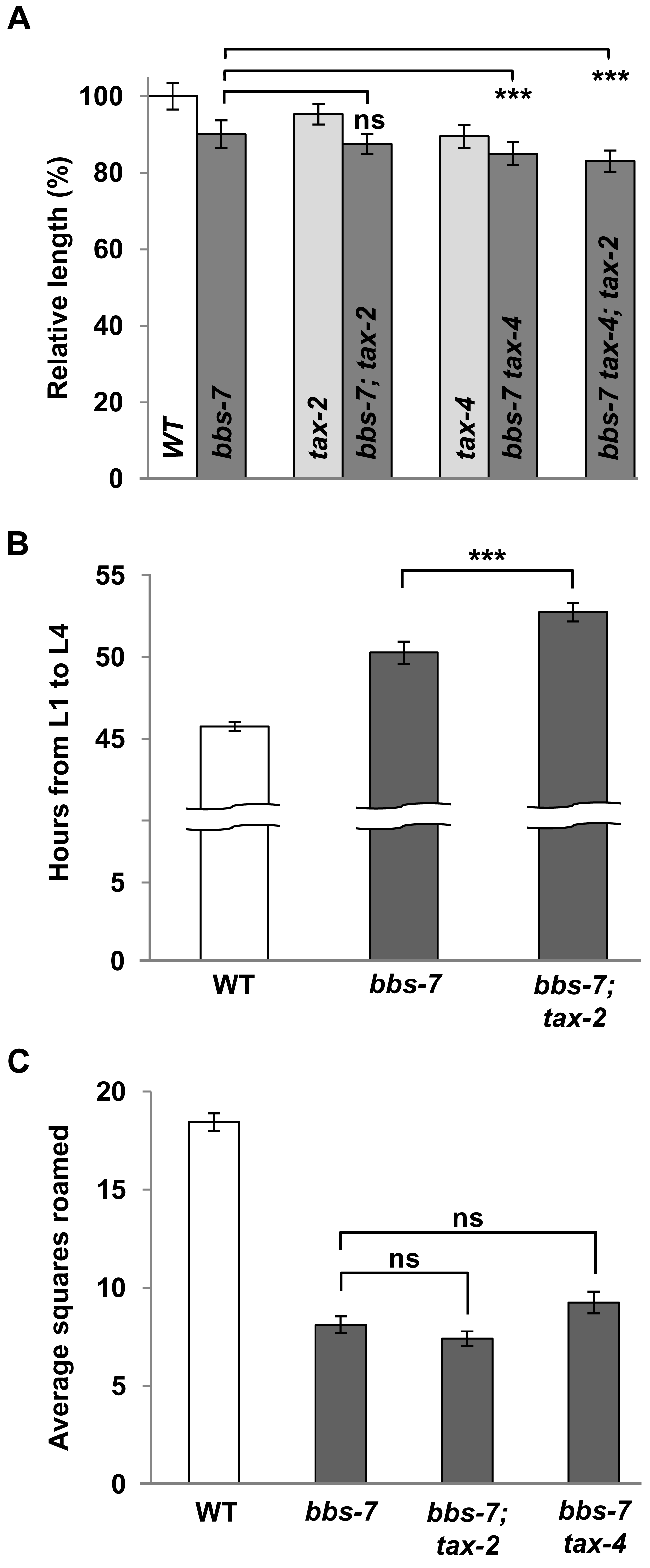

Supplement: Figure S4 — The cGMP-gated ion channel TAX-2/4 does not suppress the body size defects of bbs mutants (A) tax-2 and tax-4 failed to rescue bbs-7 mutant body size defects. ANOVA with Tukey, *** p<0.001; ns – p≥0.05 or length difference <3.5%, n≥20. Data represent mean ± SD normalized against wild-type body length. (B) Developmental timing in tax-2;bbs-7 mutants was further delayed in comparison to bbs-7 mutant populations. ANOVA, *** p<0.001, n≥50, N≥10 replicates. Data represent mean (hours) ± SD. (C) Roaming defects of tax-2;bbs-7 and tax-4 bbs-7 animals were no different compared to bbs-7 single animals. Kruskal-Wallis with Dunn's, ns – p≥0.05, n≥25, N≥2 replicates. Data represent mean squares roamed ± SEM. (TIF) [file pgen.1002335.s004.tif]

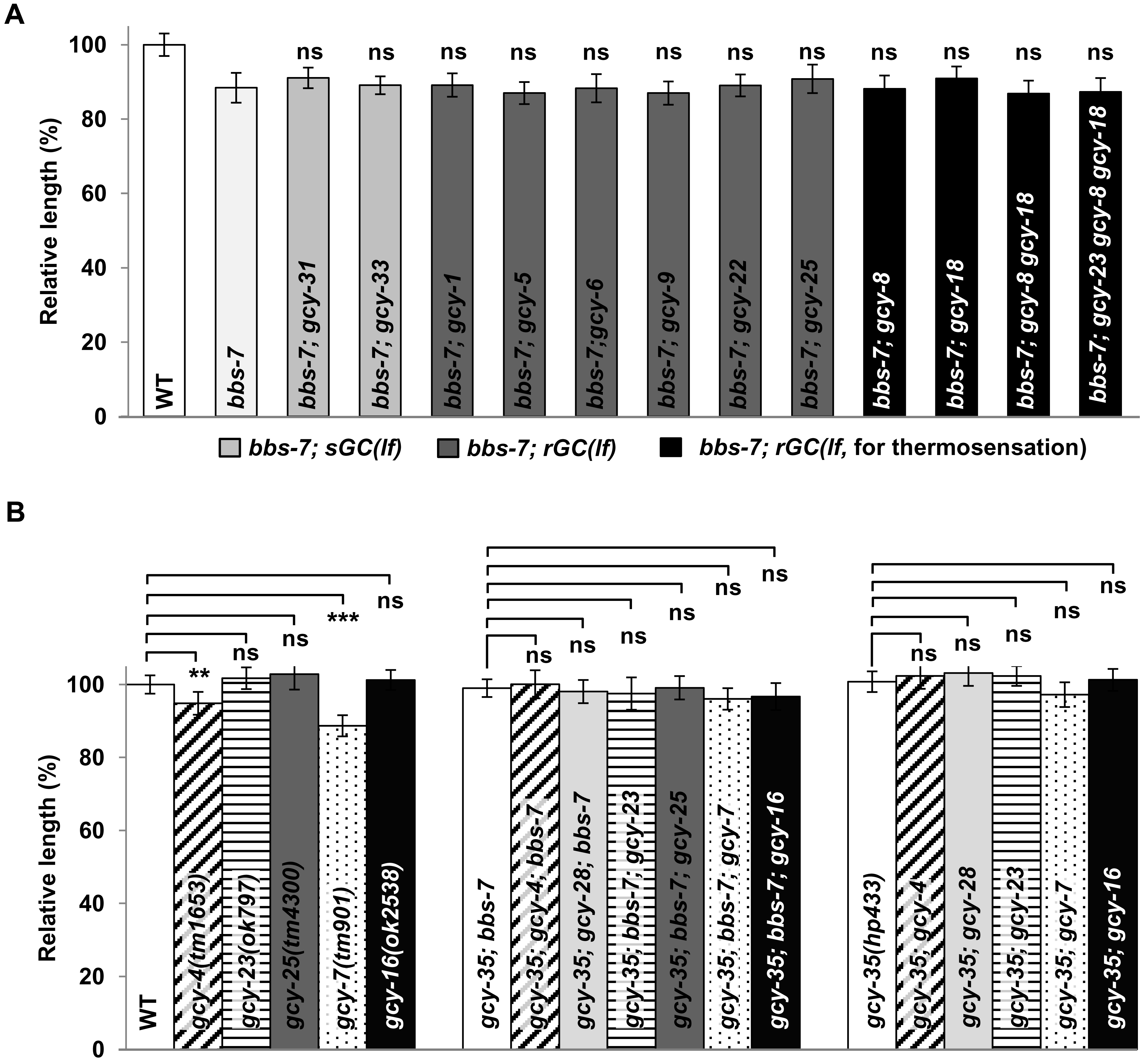

Supplement: Figure S5 — Mutations in other guanylate cyclases do not suppress bbs-7 body size defects. (A) Mutant alleles of multiple sGC genes (gray bars) and rGC genes (dark bars) did not exhibit a significant modifying effect on bbs-7 mutants. The loss of functionally redundant rGCs (gcy-8, -18, -23) [52] does not significantly modify bbs-7 body size defects either (black bars). ANOVA with Tukey, ns – p<0.05, n≥30. (B) Suppression of bbs-7 body size by the loss of gcy-35 was not significantly influenced by the loss of additional rGCs. ANOVA with Tukey, *** p<0.001; ** p<0.01; ns – p≥0.05, n≥20. All data represent mean ± SD normalized against wild-type body length. (TIF) [file pgen.1002335.s005.tif]
