# Supplementary material for: Mutations in a Guanylate Cyclase GCY-35/GCY-36 Modify Bardet-Biedl Syndrome–Associated Phenotypes in Caenorhabditis elegans
Source: PLoS Genet. 2011 Oct 13;7(10):e1002335. doi: 10.1371/journal.pgen.1002335 (PMC3192831; doi:10.1371/journal.pgen.1002335)
Supplement: Table S1 — A list of the bbs mutant alleles used in this study, outlining the type of change or deletion characterized in these strains and the resulting changes to protein translation or domains. (DOC) [file pgen.1002335.s006.doc]

**Table S1.** A list of *bbs* *C. elegans* mutants examined in this study

| **Gene** | **Allele** | **Strain** | **LG** | **Exons Deleted** | **Domains Deleted or Deletion Result** |
| --- | --- | --- | --- | --- | --- |
| *bbs-1* | *ok1111* | VC837 | I | 7,8,9 (partial) | C-terminal truncation. No known domain information |
| *bbs-2* | *gk544* | VC1168 | IV | 1 (partial), 2, 3 (partial) | Ochre stop (aa34) |
| *bbs-7* | *n1606* | MT3645 | III | 4 (Single base-pair mutation) | Opal stop (aa223) |
| *bbs-7* | *ok1351* | RB1268 | III | 6,7 | Likely loss of γ-adaptin ear domain |
| *bbs-8* | *nx77* | MX52 | V | 3 (partial), 4, 5, 6 (partial) | Likely truncation of protein and loss of TPR, Coatomer_E, Pilf, TadD, NrfG domains |
| *bbs-9* | *gk471* | VC1062 | I | 1,2,3 | Truncation of N-terminal β propeller |
