# Supplementary material for: Mutations in a Guanylate Cyclase GCY-35/GCY-36 Modify Bardet-Biedl Syndrome–Associated Phenotypes in Caenorhabditis elegans
Source: PLoS Genet. 2011 Oct 13;7(10):e1002335. doi: 10.1371/journal.pgen.1002335 (PMC3192831; doi:10.1371/journal.pgen.1002335)
Supplement: Text S1 — includes a list of strains generated and used in this study, and methods for molecular biology, phenotype analyses, statistical analyses and fluorescent microscopy. (DOC) [file pgen.1002335.s007.doc]

**Text S1:**

**Strains:** All single mutant strains were backcrossed against N2 at least 4 times.

*bbs* strains – ZM1717 *bbs-1(ok1111)I*, ZM1718 *bbs-7(ok1351)III*, ZM1719 *bbs-7(n1606)III*, ZM3709 *bbs-2(gk544)IV*, ZM3749 *bbs-9(gk471)*, MX52 *bbs-8(nx77)V,* ZM2090 *bbs-1(ok1111)I;bbs-7(ok1351)III*, ZM2091 *bbs-1(ok1111)I;bbs-8(nx77)V*, ZM2092 *bbs-7(ok1351)III;bbs-8(nx77)V*, ZM2093 *bbs-1(ok1111)I;bbs-7(ok1351)III;bbs-8(nx77)V*

*dyf* strains – CB1033 *che-2(e1033)X*, ZM5814 *che-3(e1124)I*, ZM5816 *che-3(ok1574)I*, ZM5825 *che-11(e1810)V*, ZM5821 *osm-3(p802)IV*, ZM5823 *osm-5(p813)X*, ZM5812 *osm-6(p811)V,* ZM6333 *klp-11(tm324)IV*

sGC mutants – ZM2085 *gcy-35(hp433)I*, RB906 *gcy-35(ok769)I*, RB1048 *gcy-32(ok995)V*, RB1062 *gcy-34(ok1012)V*, RB626 *gcy-37(ok384)IV*, RB564 *gcy-31(ok296)X*, CZ3715 *gcy-33(ok232)V,* AX1297 *gcy-36(db66)X*

Others – PR671 *tax-2(p671)I*, FK103 *tax-4(ks28)III*, CX7102 *qaIs2241[Pgcy-36::egl-1]X*, MT1073 *egl-4(n478)IV*, DA521 *egl-4(ad450)IV*

Combination mutants - ZM2087 *gcy-35(hp433)I; bbs-7(ok1351)III*, ZM2086 *gcy-35(hp433)I; bbs-7(n1606)III*, ZM3703 *gcy-35(ok769)I; bbs-7(ok1351)III*, ZM4059 *bbs-7(ok1351)III; gcy-36(db66)X*, ZM3861 *bbs-7(ok1351)III; gcy-32((ok995)V*, ZM3862 *bbs-7(ok1351)III; gcy-34(ok1012)V*, ZM3863 *bbs-7(ok1351)III; gcy-37(ok384)IV*, ZM5309 *bbs-7(ok1351)III; gcy-31(ok296)X*, ZM5269 *bbs-7(ok1351)III; gcy-33(ok232)V*, ZM4174 *gcy-35(hp433)I*; *bbs-7(ok1351)III; gcy-36(db66)X*, ZM3704 *gcy-35(hp433)I; bbs-8(nx77)V*, ZM3859 *gcy-35(hp433) bbs-1(ok1111)I*, ZM3705 *gcy-35(ok769)I; bbs-2(gk544)IV*, ZM3856 *gcy-35(ok769)I; bbs-8(nx77)V*, ZM3857 *gcy-35(ok769) bbs-1(ok1111)I,* ZM5274 *tax-2(p671)I; bbs-7(ok1351)III,* ZM5270 *tax-4(ks28) bbs-7(ok1351)III*, ZM4729 *gcy-35(hp433)I; egl-4(n478)IV*, ZM4727 *bbs-7(ok1351)III; egl-4(n478)IV*, ZM4730 *gcy-35(hp433)I; bbs-7(ok1351)III; egl-4(n478)IV*, ZM5272 *gcy-35(hp433)I; egl-4(ad450)IV*, ZM5273 *bbs-7(ok1351)III; egl-4(ad450)IV*, ZM5271 *gcy-35(hp433)I; bbs-7(ok1351)III; egl-4(ad450)IV*

rGC combination mutants – ZM5725 *gcy-1(tm2669)II;bbs-7(ok1351)III*, ZM5726 *gcy-4(tm1653)II;bbs-7(ok1351)III*, ZM5613 *gcy-5(ok930)II;bbs-7(ok1351)III*, ZM5727 *bbs-7(ok1351)III;gcy-6(tm1449)V*, ZM5728 *bbs-7(ok1351)III;gcy-7(tm901)V*, ZM5465 *bbs-7(ok1351)III;gcy-8(oy44)IV*, ZM5729 *bbs-7(ok1351)III;gcy-9(tm2816)X*, ZM5614 *bbs-7(ok1351)III;gcy-16(ok2538)V*, ZM5466 *bbs-7(ok1351)III;gcy-18(nj38)IV*, ZM5730 *bbs-7(ok1351)III;gcy-22(tm2364)V*, ZM5612 *bbs-7(ok1351)III;gcy-23(ok797)IV*, ZM5731 *bbs-7(ok1351)III;gcy-5(tm4300)V*, ZM5732 *bbs-7(ok1351)III;gcy-28(tm2411)I*, ZM5615 *bbs-7(ok1351)III; gyc-8(oy44) gcy-18(nj38),* ZM5733 *bbs-7(ok1351)III; gcy-23(ok797) gyc-8(oy44) gcy-18(nj38),* ZM5467 *bbs-7(ok1351)III;odr-10(n1936)X*, ZM4928 *bbs-7(ok1351)III;daf-11(m47)V*, ZM5616 *bbs-7(ok1351)III;daf-11(ks67)V*, ZM5991 *gcy-35(hp433)I;gcy-23(ok797)IV,* ZM5992 *gcy-35(hp433)I;gcy-23(ok797)IV;bbs-7(ok1351)III,* ZM5993 *gcy-35(hp433)I;gcy-4(tm1653)II*, ZM5994 *gcy-35(hp433)I;gcy-4(tm1653)II;bbs-7(ok1351)III*, ZM5996 *gcy-35(hp433)I;bbs-7(ok1351)III;gcy-25(tm4300)IV,* ZM5997 *gcy-28(tm2411)* *gcy-35(hp433)I; bbs-7(ok1351)III,* ZM5998 *gcy-35(hp433)I;gcy-16(ok2538)V,* ZM5999 *gcy-35(hp433)I;bbs-7(ok1351)III;gcy-16(ok2538)V,* ZM6000 *gcy-35(hp433)I;gcy-7(tm901)V,* ZM6001 *gcy-35(hp433)I;bbs-7(ok1351)III;gcy-7(tm901)V*

*dyf* combination mutants – ZM5817 *gcy-35(hp433)I;che-2(e1033)X*, ZM5815 *gcy-35(hp433) che-3(e1124)I*, ZM5818 *gcy-35(hp433) che-3(ok1574)I*, ZM5826 *gcy-35(hp433)I;che-11(e1810)V*, ZM5822 *gcy-35(hp433)I;osm-3(p802)IV*, ZM5824 *gcy-35(hp433)I;osm-5(p813)X*, ZM5813 *gcy-35(hp433)I;osm-6(p811)V*, ZM6334 *gcy-35(hp433)I;klp-11(tm324)IV*

**Transgenic Strains**: Extrachromosomal arrays (*hpEx* lines) were generated by co-injecting various DNA constructs at ~10-20ng/ul with co-injection markers at 10ng/ul. The following are the strains and genotypes of the *hpEx* lines used in this study:

Rescuing lines for *bbs* animals – ZM(1990, 1992) *bbs-7(n1606)III; hpEx(463,465)[bbs-7(wt)+Podr-1::GFP])*, ZM2001 *bbs-7(ok1351)III; hpEx472[bbs-7(wt)+Podr-1::GFP]*, ZM(2460-2463) *bbs-8(nx77)V; hpEx(636-639)[bbs-8(wt)+Podr-1::GFP]*, ZM(2464-2470) *bbs-1(ok1111)I; hpEx(640-646)[bbs-1(wt)+Podr-1::GFP]*, ZM(4007-4008) *bbs-2(gk544)IV; hpEx(1551-1552)[pJH1516+Podr-1::GFP]*, ZM2459 *gcy-35(hp433)I; bbs-7(n1606)III; hpEx635[bbs-7+Podr-1::GFP],* ZM5522 *bbs-2(gk544) hpIs213(Pgcy-36::GFP::BBS-2)IV*

Rescuing lines for *gcy-35(hp433)I;bbs-7(ok1351)III* animals – ZM3390 *gcy-35(hp433)I; bbs-7(ok1351)III; hpEx1251[WRM0641cB09+Podr-1]*, ZM3391 *gcy-35(hp433)I; bbs-7(ok1351)III; hpEx1252[WRM063aC10+Podr-1])*, ZM(3393-3394) *gcy-35(hp433)I;bbs-7(ok1351)III; hpEx(1256-1257)[WRM0613cC03+Podr-1]*, ZM3395 *gcy-35(hp433)I; bbs-7(ok1351)III; hpEx1262[WRM0641cB09/MluI+Podr-1])*.

Cell-specific rescue lines for *gcy-35(hp433)I;bbs-7(ok1351)III* animals – ZM(3629-3632) *gcy-35(hp433)I;bbs-7(ok1351)III; hpEx(1360-1363)[Pgcy-36::GCY-35(non-isoprenylated)+Podr-1]*, ZM(3633-3635) *gcy-35(hp433)I;bbs-7(ok1351)III; hpEx1364-1366[Plad-2::GCY-35(isoprenylated)+Podr-1]*, ZM(3747-3748) *gcy-35(hp433)I; bbs-7(ok1351)III; hpEx(1367-1368)[pJH1374+Podr-1]*, ZM(4062-4063) *gcy-35(hp433)I; bbs-7(ok1351)III; hpEx(1568-1569)[pJH1493+Podr-1]*

Transgenic lines to examine the subcellular localisation of GCY-35 and GCY-36 – ZM4064 *gcy-36(db66)X; hpEx1570[Pgcy-36::GFP::GCY-36+Podr-1::GFP]*, ZM4065 *bbs-7(ok1351)III; gcy-36(db66)X; hpEx1571[Pgcy-36::GFP::GCY-36+ Podr-1::GFP]*, ZM5540 *gcy-35(hp433)I; hpEx2312[pJH1867+Podr-1::GFP]*, ZM(5538-5539) *gcy-35(hp433)I; bbs-7(ok1351)III; hpEx(2310-2311)[pJH1867+Podr-1::GFP],* ZM5529 *hpIs215[Pgcy-36::GFP::GCY-36](X*), ZM5530 *bbs-7(ok1351)III; hpIs215(X)*

EGL-4 gain of function expression lines – ZM(5534-5536) *gcy-35(hp433)I; bbs-7(ok1351)III; hpEx(2306-2308)[Pgcy-36::egl-4(ad450)+Podr-1::GFP]*, ZM5537 *N2; hpEx2309[Pgcy-36::egl-4(ad450)+Podr-1::GFP]*

**Molecular biology:** Genomic rescues of *bbs-1*, *bbs-7*, and *bbs-8* used PCR products of each respective genomic sequence, including 2080, 1262, and 531bp of 5’ upstream sequence respectively. Rescue of *bbs-2* animals used *bbs-2* cDNA (F20D12.3 ordered from OpenBiosystems, catalog # OCE1182-7243861) driven by an F25B3.3 (*rgef-1*) promoter sequence of 3444bp. *gcy-35* rescues were carried out with clones *Pgcy-36::GCY-35* (*non-isoprenylated)* [1] and *Plad-2::GCY-35* (*isoprenylated)* [2] courtesy of Dr. Cornelia Bargmann. Promoters for these two plasmids were 1095bp and 4013bp respectively and exchanged using FseI and AscI sites to generate *pJH1374 (Pgcy-36::GCY-35 isoprenylated)* and *pJH1390 (Plad-2::GCY-35 non-isoprenylated)*. *pJH1493 (Pflp-8::GCY-35 isoprenylated)* was created by insertion of a FseI/AscI tagged, 2.3kb sequence 5’ of the *flp-8* start site PCR’d from N2 genomic DNA (p3548: ATTggccggccTCAGAAACCCCGATTCAAAC, p3549: gtcGGCGCGCCTTTCTACTTG AAAAGTGTGGAC).

Subcellular localisation of GCY-36 was analysed using a vector of *Pgcy-36::GFP::GCY-36* [3] courtesy of Dr. Mario de Bono that included 2021bp of upstream promoter sequence. *pJH1867 (Pgcy-36::GFP::GCY-35)* was constructed by replacing GCY-36 in the above construct with a subcloned GCY-35 from *pJH1374* using p3639 SfoI-tagged (ATTGGCGCCacaATGTTCGGCT GGATTCACG) and p3640 HpaI-tagged (GTCgttaacttaAGAAATTGTGCAAGTCG) primers.

**Phenotype analyses of *C. elegans* wild-type and mutant animals**

**DiI uptake** - DiI assays were performed on mixed populations of strains as previously described [4]. *C. elegans* were washed twice in M9 buffer and resuspended in 400ul M9 with 2ng of DiI. Samples were incubated 2-3 hours before washing three times with M9 buffer. Worms were allowed to recover overnight before scoring for uptake phenotypes. Uptake was scored as positive in an animal if DiI staining was observed in amphid, phasmid or both sets of neurons on a minimum of 20 animals per strain. Fisher’s Exact test was used to compare rescues between wildt-type and *bbs* strains as well as between *bbs* and *bbs* rescue strains.

**Body size assessment** - Three gravid adult animals per strain were allowed to lay eggs for 24-30 hours before being removed from each plate. F1 animals were allowed to grow for an additional 48 hours. Approximately 20-40 L4 animals were measured for each strain. L4 animals were allowed to grow 16-22 hours before measurement for adult data. Some populations were measured again 48 hours later to complete growth curve analysis.

For body size measurement, images were captured on a Zeiss Stemi SVII dissecting microscope at 50X magnification using a Zeiss Axiocam digital camera at a 1388x1040 resolution. These images were then analysed using Axiovision software to measure length (cubic splines) and/or width of individual animals.

Statistical analyses were completed using ANOVA with the Tukey post-hoc test to compare between multiple groups; *** p<0.001 ** p<0.01, * p<0.05, and ns p≥0.05. Statistical significance is only noted for a body size difference of >3.5% of wild-type length. Data on all graphs represent mean ± standard deviation relative to wild-type body length.

**Developmental timing** - The developmental timing assay was modified from [5]. Briefly, eggs were collected from gravid adults and hatched in 7.5mL M9 for 20-22 hours. Synchronized L1 larvae were centrifuged again and animals were deposited onto multiple OP50-seeded NGM plates at about 50-80 animals per plate. Animals were grown at 20ºC for ~42 hours before being assessed for developmental staging based on described anatomical landmarks for L4 larvae at 30-minute intervals. Each plate was analysed as a single population, with at least 10 replicate plates per strain. The ANOVA with Tukey post-hoc test was used for statistical analyses; *** p<0.001 ** p<0.01, * p<0.05, and ns p≥0.05 in comparing between strains.

**Roaming assay** – Animals at the L4 stage were transferred individually to the centre of 35mm seeded plates. Individual animals were removed 18 hours later and their tracking pattern was analysed using a 5mm x 5mm grid. Inclusion criteria for grids were any bacterial squares that the animals had traversed in the given time. For each strain a minimum of 25 animals was examined. Statistical analyses were completed using the Kruskal-Wallis non-parametric test with Dunn’s post-hoc to compare between multiple groups; *** p<0.001 ** p<0.01, * p<0.05, and ns p≥0.05. Data on all graphs represent mean ± standard error of mean (SEM) for squares traversed.

**Fluorescence Microscopy**

Animals were bleach synchronized and examined at various stages including at L3, L4 and 12-20 hours later. Animals were immobilised on 2-5% dry agarose imaging pads, with 2ul of M9 solution. All images were acquired on a Zeiss Axioskop 2 fluorescent microscope at 63x magnification equipped with a CCD camera.

**Supplemental References**

1. Gray JM, Karow DS, Lu H, Chang AJ, Chang JS, et al. (2004) Oxygen sensation and social feeding mediated by a *C. elegans* guanylate cyclase homologue. Nature 430: 317-322.

2. Chang AJ, Chronis N, Karow DS, Marletta MA, Bargmann CI (2006) A distributed chemosensory circuit for oxygen preference in *C. elegans*. PLoS Biol 4: e274.

3. Cheung BH, Arellano-Carbajal F, Rybicki I, de Bono M (2004) Soluble guanylate cyclases act in neurons exposed to the body fluid to promote *C. elegans* aggregation behavior. Curr Biol 14: 1105-1111.

4. Uchida O, Nakano H, Koga M, Ohshima Y (2003) The *C. elegans* che-1 gene encodes a zinc finger transcription factor required for specification of the ASE chemosensory neurons. Development 130: 1215-1224.

5. Sulston JE, A. HJ (1988) Methods. The Nematode *Caenorhabditis elegans*. New York: Cold Spring Harbor Laboratory Press. pp. 587-606.
